# Ca^2+^ pushes and pulls energetics to maintain ATP balance in atrial cells: computational insights

**DOI:** 10.3389/fphys.2023.1231259

**Published:** 2023-07-17

**Authors:** Noam Keidar, Noa Kirschner Peretz, Yael Yaniv

**Affiliations:** Laboratory of Bioenergetic and Bioelectric Systems, Biomedical Engineering Faculty, Technion-Israel Institute of Technology (IIT), Haifa, Israel

**Keywords:** atria, energetic balance, ATP, computational modeling, ATP supply

## Abstract

To maintain atrial function, ATP supply-to-demand matching must be tightly controlled. Ca^2+^ can modulate both energy consumption and production. In light of evidence suggesting that Ca^2+^ affects energetics through “push” (activating metabolite flux and enzymes in the Krebs cycle to push the redox flux) and “pull” (acting directly on ATP synthase and driving the redox flux through the electron transport chain and increasing ATP production) pathways, we investigated whether both pathways are necessary to maintain atrial ATP supply-to-demand matching. Rabbit right atrial cells were electrically stimulated at different rates, and oxygen consumption and flavoprotein fluorescence were measured. To gain mechanistic insight into the regulators of ATP supply-to-demand matching in atrial cells, models of atrial electrophysiology, Ca^2+^ cycling and force were integrated with a model of mitochondrial Ca^2+^ and a modified model of mitochondrial energy metabolism. The experimental results showed that oxygen consumption increased in response to increases in the electrical stimulation rate. The model reproduced these findings and predicted that the increase in oxygen consumption is associated with metabolic homeostasis. The model predicted that Ca^2+^ must act both in “push” and “pull” pathways to increase oxygen consumption. In contrast to ventricular trabeculae, no rapid time-dependent changes in mitochondrial flavoprotein fluorescence were measured upon an abrupt change in workload. The model reproduced these findings and predicted that the maintenance of metabolic homeostasis is due to the effects of Ca^2+^ on ATP production. Taken together, this work provides evidence of Ca^2+^ “push” and “pull” activity to maintain metabolic homeostasis in atrial cells.

## 1 Introduction

The atria conduct the electrical activity from the sinoatrial node to the heart’s ventricles and both passively and actively enhance ventricular diastolic filling. High and rapidly fluctuating metabolic rates are necessary to perform these electrical and mechanical tasks (Balaban, 2012), thus ATP supply-to-demand matching is crucial. Although mechanisms that match ventricular ATP supply to demand have been investigated since the early 1950s, the identity and the specific targets of the control mechanisms, even under normal conditions, remain controversial (Yaniv et al., 2010). In addition, regulation of atrial energetics is not well understood.

Mitochondria are the main producers of ATP in atrial cells, and both Ca^2+^ and ADP have been suggested as key regulators of matching respiratory to energy supply to varying demands (Mason et al., 2020); yet their targets remain to be determined (Liberopoulos, 2013). ADP activates enzymes in the Krebs cycle, and thereby “pushes” the respiratory flux toward ATP generation (Harris and Das, 1991). In parallel, ADP controls ATP synthase by its availability, and thereby “pulls” ATP production by increasing respiratory flux (Chance and Williams, 1955). Activation of ATP synthase facilitates redox flux through the electron transport chain and increases ATP production. While Ca^2+^ is generally thought to act as a “push” control over ATP production (Harris and Das, 1991), by activating metabolite flux and enzymes in the Krebs cycle to push the redox flux, accumulating evidence suggests its role as a “pull” regulator (Territo et al., 2000). Whether Ca^2+^ control of metabolism works via both “pull” and “push” mechanisms in atrial cells under physiological conditions is not known. Moreover, while metabolic homeostasis in response to increased workload was shown in the heart (Balaban, 2012), it remains to be determined whether it is maintained in atrial cells in response to electrical stimulation.

To gain mechanistic insights into the regulators of such matching in atrial cells, this work constructed a computer model that integrated models of electrophysiology (Lindblad et al., 1996), Ca^2+^ cycling (Lindblad et al., 1996; Aslanidi et al., 2009) and force (Yaniv et al., 2006), to describe ATP demand, with a model of mitochondrial Ca^2+^ (Nguyen et al., 2007) and a modified model of mitochondrial energy metabolism (Cortassa et al., 2006). The integrated model was validated by conducting experiments measuring oxygen consumption and flavoprotein activity in response to increased electrical stimulation rates.

We hypothesized that: i) Ca^2+^ is an important regulator of ATP supply-to-demand matching, ii) Ca^2+^ both “pulls” and “pushes” ATP production and iii) metabolic homeostasis is maintained in atrial cells during increased electrical stimulation or abrupt changes in workload.

By combining experimental and computational results, we found ATP supply only matched the demand when Ca^2+^ both “pulled” and “pushed” ATP. Moreover, no rapid time-dependent changes in mitochondrial flavoprotein fluorescence level were observed in response to an abrupt change in workload, due to Ca^2+^ ion regulation of cell bioenergetics.

## 2 Materials and methods

### 2.1 Animal use

Animals were treated in accordance with the Technion Ethics Committee. The experimental protocols were approved by the Animal Care and Use Committee of the Technion (Ethics numbers: IL-118-10-13 and IL-001-01-19).

### 2.2 Atrial cell isolation

Hearts were isolated from healthy male New Zealand white rabbits weighing 2.3–2.7 kg. Each rabbit was weighed and then sedated with an intramuscular injection of ketamine (0.1 ml/kg) and xylazine (0.1 ml/kg). An intravenous cannula was inserted in the rabbit’s ear for delivery of 200 mg/ml sodium pentobarbital diluted with heparin. The adequacy of the anesthesia was evaluated by observing the loss of reflexes in the eye and foot. The atrial cell isolation procedure is described in (Kirschner Peretz et al., 2017).

### 2.3 Cytosolic Ca^2+^ measurements

Ca^2+^ cycling into and out of the atrial cell cytosol was measured with Fluo-4 AM (ThermoFisher Scientific), as previously described (Davoodi et al., 2017). Data from (Davoodi et al., 2017; Kirschner Peretz et al., 2017) were reanalyzed on a custom-made guided user interface (GUI) programmed in MATLAB (Davoodi et al., 2017).

### 2.4 Electrical stimulation

Cytosolic Ca^2+^ dynamics, flavoprotein autofluorescence and oxygen consumption following electrical stimulation at 1–3 Hz were measured. Electrical fields were created by a pair of platinum electrodes (0.008″bare wire, A-M Systems) glued to a custom-made chamber top.

### 2.5 Flavoprotein autofluorescence measurements

The autofluorescence of mitochondrial flavoprotein of atrial cells was imaged, at different electrical stimulation frequencies (quiescent, 0.25, 1, 2, and 3 Hz), at 37°C ± 0.5°C, under an inverted fluorescence microscope (Zeiss Observer Z1) with a 40x/1.4 N.A oil immersion lens and a 445 nm light-emitting diode (LED). Images were captured at 2 frames per second.

### 2.6 Oxygen consumption

Oxygen consumption by either quiescent or electrically stimulated (1, 2 or 3 Hz) atrial cell suspensions was measured using Clark-type electrodes (MT200, Strathkelvin Instruments Ltd.). For the oxygen chamber, a custom-sealed plunger that included platinum wires was designed, as previously described (Yaniv et al., 2011). Atrial cell suspensions were centrifuged at 1000 RPM for 10 min, and the supernatant was aspirated. The resuspended cells were incubated in fresh HEPES solution containing (in mM): NaCl 140, KCl 5.4, HEPES 5, glucose 10, MgCl_2_ 2, and CaCl_2_ 1 (pH 7.4 with NaOH). The cell suspension was divided into two equal aliquots, one of which was subjected to electrical stimulation and the second served as a control. The atrial cell suspensions were stirred gently under quiescent conditions in HEPES buffer for 2 min at 36°C, and then electrically stimulated at 1, 2 and 3 Hz for 1 min and again maintained under quiescent conditions for 1 min. Following measurement of oxygen consumption, total protein concentration (BCA™ Protein Assay) and the number of viable cells in the cell suspension were determined. Oxygen consumption of the atrial cells was normalized to the protein concentration.

### 2.7 Statistics

All experiments were performed on cells from at least three rabbits. All data are presented as mean ± SD. Comparisons were made using a one-way repeated measurement ANOVA test with *p* < 0.05 taken to indicate statistical significance.

### 2.8 The numerical model general approach

**FIGURE 1:**
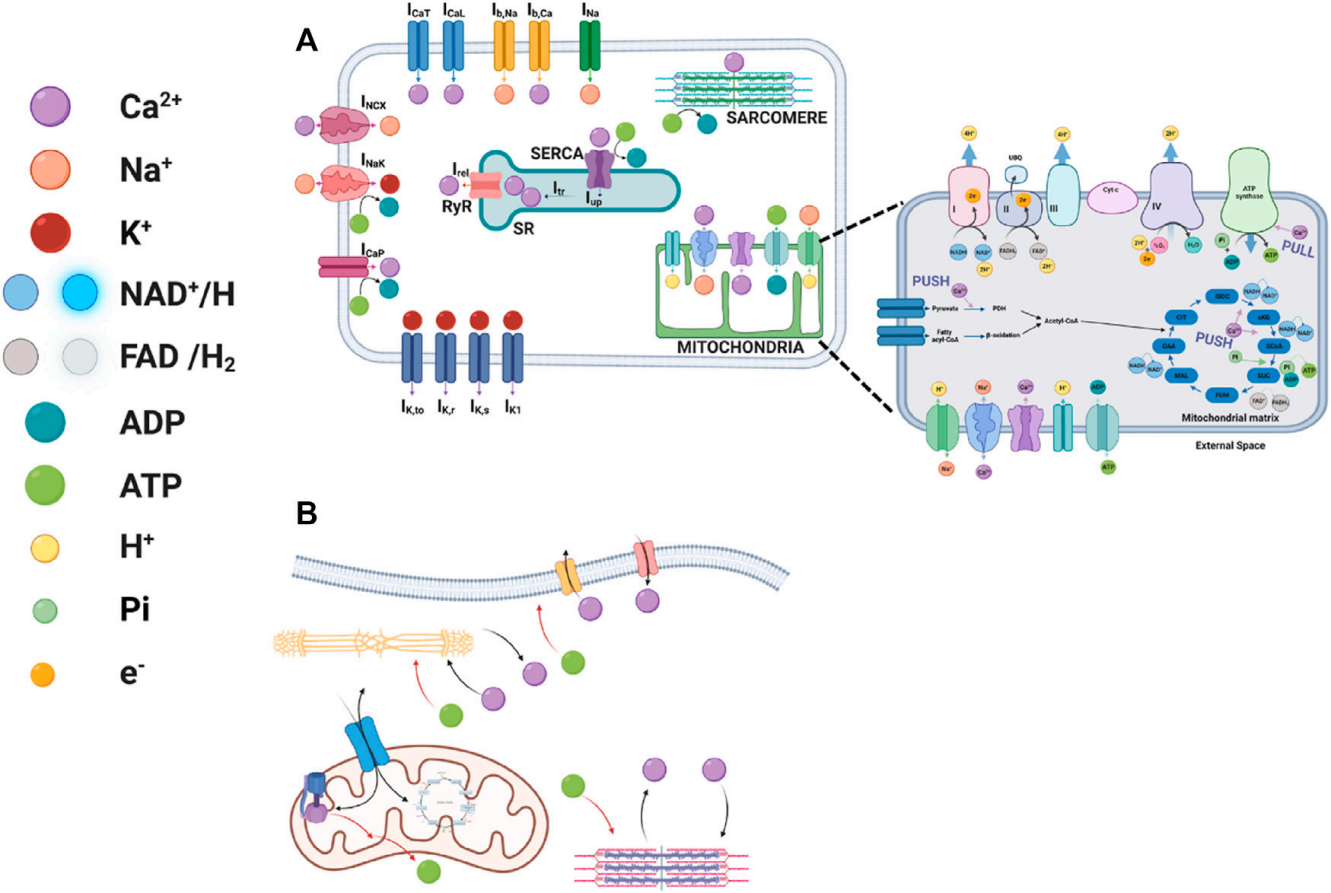
Schematic illustrations of excitation-contraction-energetics coupling. **(A)** The interplay between ionic channels and exchangers to the myofilaments, sarcoplasmic reticulum Ca^2+^ cycling proteins, and basal state ATP demand and the interplay between Krebs cycle proteins, pyruvate dehydrogenize, mitochondrial complexes, channels and exchangers with Ca^2+^ ions in atrial cells. **(B)** The interconnected signaling mediating the different models. Created with BioRender.com.

The model describing the atrial membrane potential, ionic currents, Ca^2+^ cycling, force generation and energetics (Figure 1A) was based on rabbit atrial excitation-contraction modeling (Lindblad et al., 1996) with a model of troponin-myosin concentration (Yaniv et al., 2006), whose parameters were modified to fit atrial force data (Beyer et al., 1986; Komai and Rusy, 1990). The energetics model was a modified version of a model described by Cortassa et al. (Cortassa et al., 2006) and included mitochondrial Ca^2+^ dynamics that was based on an earlier report (Nguyen et al., 2007). The interconnection between the models is described in Figure 1B. Our suggestion for the metabolic steps affected by the Ca^2+^ push and pull mechanisms are shown in the mitochondrion part of Figure 1A.

For conciseness, only novel assumptions and modifications in the model equations are described here, while the full model and the modified parameters is described in the supplement.

#### 2.8.1 Assumptions

(i) The ATP consumption by Na^+^-K^+^ ATPase and the membranal Ca^2+^ pump is low, thus we used the original equation described in (Lindblad et al., 1996).
(ii) The force generation can be described by a four-state rather than a two-state kinetics (Yaniv et al., 2006) model.
(iii) Similar to NADH, FADH_2_ concentration is not constant (Harris and Das, 1991).
(iv) As the reducing reagents, FADH_2_ and NADH are created in the TCA cycle in a 1:4 ratio (FADH_2_:NADH), and we assume that their consumption by the electron transfer chain follows the same ratio (Harris and Das, 1991).
(v) Regulation of oxidative phosphorylation by the cellular energy demands can be modeled using a push and pull mechanism (Balaban, 2009).
(vi) Ca^2+^ regulates the activity of F_1_F_o_-ATPase (Balaban, 2012).

#### 2.8.2 Modification in the model

An ATP-dependent variable was added to the original uptake current of Ca^2+^ into the sarcoplasmic reticulum (SR) equation in (Lindblad et al., 1996):

$$I_{up}=I_{up,\max}∙\frac{{\left[ATP\right]}_{i}}{7.977}\frac{\frac{{\left[Ca^{2+}\right]}_{i}}{K_{cy,Ca}}-\frac{K_{xcs}^{2}{\left[Ca^{2+}\right]}_{up}}{K_{sr,Ca}}}{\frac{{\left[Ca^{2+}\right]}_{i}+K_{cy,Ca}}{K_{cy,Ca}}+\frac{K_{xcs}\left({\left[Ca^{2+}\right]}_{up}+K_{sr,Ca}\right)}{K_{sr,Ca}}}$$ (1)

where $I_{up,\max}$ is the maximal calcium uptake current into the SR, $K_{cy,Ca}$ is the equilibrium binding $Ca^{2+}$ concentration on the cytosolic side [ ${\left[Ca^{2+}\right]}_{i}$ ) $K_{sr,Ca}$ is the equilibrium binding $Ca^{2+}$ concentration in the uptake compartment of the SR side, $K_{xcs}$ is a translocation constant and ${\left[Ca^{2+}\right]}_{up}$ is the Ca^2+^ concentration in the uptake compartment of the SR.

Because the force is described by four state that are affected by Ca^2+^, instead of on and off kinetics, the amount of bound Ca^2+^ to the tropomyosin is described by:

$$\frac{dO_{TnCa}}{dt}=F_{kl}{\left[Ca^{2+}\right]}_{i}\left(1-A-TT-U\right)+F_{kl}{\left[Ca^{2+}\right]}_{i}U-TTK_{-1}-A\;K_{-1}$$ (2)

where K_-1_ describes the dependence of Ca^2+^ affinity on the number of strong cross-bridges and $F_{kl}$ is the rate constant of calcium binding to low-affinity troponin sites. A is the density of regulatory units with bound Ca^2+^ and adjacent weak cross-bridges. TT is the density of regulatory units with bound Ca^2+^ and adjacent strong cross-bridge. U is the density of regulatory units without bound Ca^2+^ but with adjacent strong cross-bridges.

We modified the energetics equation in (Cortassa et al., 2006) to include regulation by mitochondrial Ca^2+^ ([Ca^2+^]_m_), as suggested by (Harris and Das, 1991). Thus, the rate of pyruvate dehydrogenase cam be described by:

$$V_{PDH}=k_{PDH}∙C_{PYR}∙\left(\frac{{\left[Ca^{2+}\right]}_{m}}{{\left[Ca^{2+}\right]}_{m}+k_{CaAcCoA}}\right)$$ (3)

where $k_{PDH}$ is the catalytic constant of pyruvate dehydrogenase (PDH), $C_{PYR}$ is the pyruvate concentration and $k_{CaAcCoA}$ is the Michaelis constant representing the regulatory effect of Ca^2+^ on PDH.

The respiration-driven proton pump is based on (Cortassa et al., 2006), with the modification indicated below:

Mitochondrial NAD^+^ is assumed to be conserved according to the following relation:

$$\left[NAD\right]=C_{PN}-\left[NADH\right]$$ (4)

where $C_{PN}$ is the total sum of mitochondrial pyridine nucleotides.

$$A_{resNADH}=\frac{RT}{F}\ln\left(K_{res}\sqrt{\frac{\left[NADH\right]}{\left[NAD\right]}}\right)$$ (5)

$$A_{resFLV}=\frac{RT}{F}\ln\left(K_{res}\sqrt{\frac{\left[{FADH}_{2}\right]}{\left[FAD\right]}}\right)$$ (6)

$$\left[FAD\right]={Tot}_{FAD}-\left[{FADH}_{2}\right],$$ (7)

where $K_{res}$ is the equilibrium constant of respiration, $\left[{FADH}_{2}\right]$ is the concentration of reduced FAD, $\left[FADH\right]$ is the concentration of oxidized FAD and Tot_FAD_ is the total concentration of FADH_2_ and FAD.

As the reducing reagents, FADH_2_ and NADH are created in the TCA cycle in a 1:4 ratio (FADH_2_:NADH) and we assume that their consumption by the electron transfer chain follows the same ratio. This means that 1/5 of oxygen consumption is attributed to FADH_2_-fueled and 4/5 to NADH-fueled electron transfer cycles:

$$A_{res}=\frac{4}{5}A_{res\_NADH}+\frac{1}{5}A_{res\_FLV}$$ (8)

In a modification of the original formulation in (Cortassa et al., 2006), it is also considered that the complex II electrons are input by SUC through FADH_2_ to the respiratory chain:

$$A_{res\left(F\right)}=\frac{RT}{F}\ln\left(K_{res\left(F\right)}\sqrt{\frac{\left[{FADH}_{2}\right]}{\left[FAD\right]}}\right)$$ (9)

$$V_{He\left(F\right)}=4\rho^{res\left(F\right)}\frac{\left(r_{a}\exp\left(\frac{A_{res\left(F\right)}F}{RT}\right)-\left(r_{a}+r_{b}\right)\exp\left(\frac{g6F∆{µ}_{H}}{RT}\right)\right)}{\left(\left(1+r_{1}\exp\left(\frac{A_{res\left(F\right)}F}{RT}\right)\right)\exp\left(\frac{6F∆\Psi_{B}}{RT}\right)+\left(r_{2}+r_{3}\exp\left(\frac{A_{res\left(F\right)}F}{RT}\right)\right)\exp\left(\frac{g6F∆{µ}_{H}}{RT}\right)\right)}$$ (10)

The flux of protons driven by FADH_2_ oxidation (V_He(F)_) has the same form as V_He_, except for the adjustment of the redox potential and the H^+^ stoichiometry. $\rho^{res\left(F\right)}$ is the concentration of electron carriers (respiratory complexes II-III-IV) and $K_{res\left(F\right)}$ is the equilibrium constant of FADH_2_ oxidation.

The regulation of oxidative phosphorylation by the cellular energy demands is modeled using a push and pull mechanism. ADP activates enzymes in the Krebs cycle, which “pushes” the respiratory flux toward ATP generation (Harris and Das, 1991). In parallel, ADP controls ATP synthase by its availability, thereby “pulling” ATP production by increasing the respiratory flux (Chance and Williams, 1955):

$$pull=\frac{V_{ATPase}}{k_{ATPase}}$$ (11)

$$V_{O2}=\rho^{res}pull\frac{\left(\left(r_{a}+r_{c1}\exp\left(\frac{6F∆\Psi_{B}}{RT}\right)\right)\exp\left(\frac{A_{res}F}{RT}\right)-r_{a}\exp\left(\frac{g6F∆{µ}_{H}}{RT}\right)+r_{c2}\exp\left(\frac{A_{res}F}{RT}\right)\exp\left(\frac{g6F∆{µ}_{H}}{RT}\right)\right)}{\left(\left(1+r_{1}\exp\left(\frac{A_{res}F}{RT}\right)\right)\exp\left(\frac{6F∆\Psi_{B}}{RT}\right)+\left(r_{2}+r_{3}\exp\left(\frac{A_{res}F}{RT}\right)\right)\exp\left(\frac{g6F∆{µ}_{H}}{RT}\right)\right)}$$ (12)

where $r_{c1}$ and $r_{c2}$ are the sum of products of rate constants, and k_ATPase_ is defined as the coupling coefficient representing the pull effect of the ATP synthase on the activity of the electron transfer chain.

According to the concept of respiratory control, mitochondrial function is governed by the availability of ADP and P_i_. The chemiosmotic hypothesis dictates that ΔΨ_m_ is lowered by an H^+^ influx, which drives the production of ATP by F_1_F_o_-ATPase.

$$V_{ATPase}=-{30∙\rho }^{F1}\frac{\left(\left(10^{2}p_{a}+p_{c1}\exp\left(\frac{3F∆\Psi_{B}}{RT}\right)\right)\exp\left(\frac{{FA}_{F1}}{RT}\right)-\left(p_{a}\exp\left(\frac{3F∆{µ}_{H}}{RT}\right)+p_{c2}\exp\left(\frac{{FA}_{F1}}{RT}\right)\exp\left(\frac{3F∆{\Psi µ}_{H}}{RT}\right)\right)\right)}{\left(\left(1+p_{1}\exp\left(\frac{{FA}_{F1}}{RT}\right)\exp\left(\frac{3F∆\Psi_{B}}{RT}\right)\right)+\left(p_{2}+p_{3}\exp\left(\frac{{FA}_{F1}}{RT}\right)\right)\exp\left(\frac{3F∆{µ}_{H}}{RT}\right)\right)}∙\left(1-\exp\left(-\frac{{Ca}_{m}}{KCaATP}\right)\right)$$ (13)

$$V_{Hu}=-3\rho^{F1}\frac{\left(10^{2}p_{a}\left(1+\exp\left(\frac{{FA}_{F1}}{RT}\right)\right)-\left(p_{a}+p_{b}\right)\exp\left(\frac{3F∆{µ}_{H}}{RT}\right)\right.}{\left(\left(1+p_{1}\exp\left(\frac{{FA}_{F1}}{RT}\right)\right)\exp\left(\frac{3F∆\Psi_{B}}{RT}\right)+\left(p_{2}+p_{3}\exp\left(\frac{{FA}_{F1}}{RT}\right)\right)\exp\left(\frac{3F∆{µ}_{H}}{RT}\right)\right)}$$ (14)

$$A_{F1}=\frac{RT}{F}\ln\left(K_{F1}\frac{{\left[ATP\right]}_{m}}{{\left[ADP\right]}_{m}Pi}\right)$$ (15)

$${\left[ATP\right]}_{m}=C_{A}-{\left[ADP\right]}_{m}$$ (16)

where $p_{a}$ , $p_{b}$ , $p_{c1}$ , $p_{c2}$ , $p_{1}$ , $p_{2}$ and $p_{3}$ are the sum of products of rate constants, $\rho^{F1}$ is the F_1_F_0_-ATPase concentration, $K_{F1}$ is the equilibrium constant of ATP hydrolysis, Pi is the inorganic phosphate concentration and $C_{A}$ is the total sum of mitochondrial adenine nucleotides.

Experimental data (see results section) of Ca^2+^ transients, oxygen consumption and flavoprotein at a 1 Hz stimulus frequency were used as input for the computational model, and the values predicted at 2 Hz and 3 Hz stimulus frequencies were compared to the experimental results.

#### 2.8.3 Modeling the “push” and “pull” mechanisms

To eliminate the “pull” mechanism, the effect of Ca^2+^ on ATP synthase was withheld by fixing the mitochondrial Ca^2+^ to its initial condition in the $V_{ATPase}$ equation, so only the ADP “pull” played a role. To shut down the “push” mechanism, the mitochondrial Ca^2+^ was fixed at a constant value (its initial condition) throughout the model, which canceled the Ca^2+^ “push” effect on the TCA cycle and ATP synthase. In addition, the coupling variable between ATP synthase activity and the electron transfer chain oxygen consumption was fixed to its initial value, which eliminated “pull” entirely.

#### 2.8.4 ATP consumption

ATP consumption was calculated as the sum of the three main energy-consuming processes in the cell: active membrane transporters, Ca^2+^ intake by the SR and sarcomere contractile elements. For the membrane component, the Na^+^-K^+^ ATPase and the membrane Ca^2+^ pump were considered:

$${\left.\frac{d\left[ATP\right]}{dt}\right|}_{membranal}=\frac{\left(I_{NaK}+I_{Cap}\right)A_{cap}}{V_{myo}F}$$ (17)

where $I_{NaK}$ and $I_{Cap}$ are the currents per unit area, $A_{cap}$ is the capacitive cell surface area, $V_{myo}$ is the myocyte volume and F is the Faraday constant.

ATP consumption by the SR is correlative to Ca^2+^ uptake by the SR:

$${\left.\frac{d\left[ATP\right]}{dt}\right|}_{SR}=\frac{0.5I_{up}}{V_{myo}F}$$ (18)

where $I_{up}$ is the SR uptake current, $V_{myo}$ is the myocyte volume and F is the Faraday constant. The current is multiplied by 0.5 to account for the stoichiometry of the calcium pump reaction.

The sarcomere energy consumption is calculated as part of the sarcomere model, in Eq. 112 in the supplement:

$${\left.\frac{d\left[ATP\right]}{dt}\right|}_{sarcomere}=V_{AM}$$ (19)

The total energy consumption is the sum of the three:

$$\frac{d\left[ATP\right]}{dt}={\left.\frac{d\left[ATP\right]}{dt}\right|}_{membranal}+{\left.\frac{d\left[ATP\right]}{dt}\right|}_{SR}+{\left.\frac{d\left[ATP\right]}{dt}\right|}_{sarcomere}$$ (20)

### 2.9 Numerical methods

The software was run in MATLAB (The MathWorks, Inc., Natick, MA, United States). Numerical integration was performed using the MATLAB ode23tb stiff solver. Impulse stimulations were modeled as boundary conditions and the ODE was solved on the intervals between impulses. A 1-s quiescent window was added between stimulation rates to ensure the completion of the last beat before initiation of a new one. Computations were performed on an Intel(R) Core(TM) i7-8550U CPU @ 1.80 GHz machine with 16 GB RAM. Upon publication, the source code of the numerical model will be available at http://bioelectric-bioenergetic-lab.net.technion.ac.il and on https://github.com/yyLabPhysAI/energetics-atrium.

## 3 Results

### 3.1 Increase in electrical stimulus frequency increases ATP supply and demand

**FIGURE 2:**
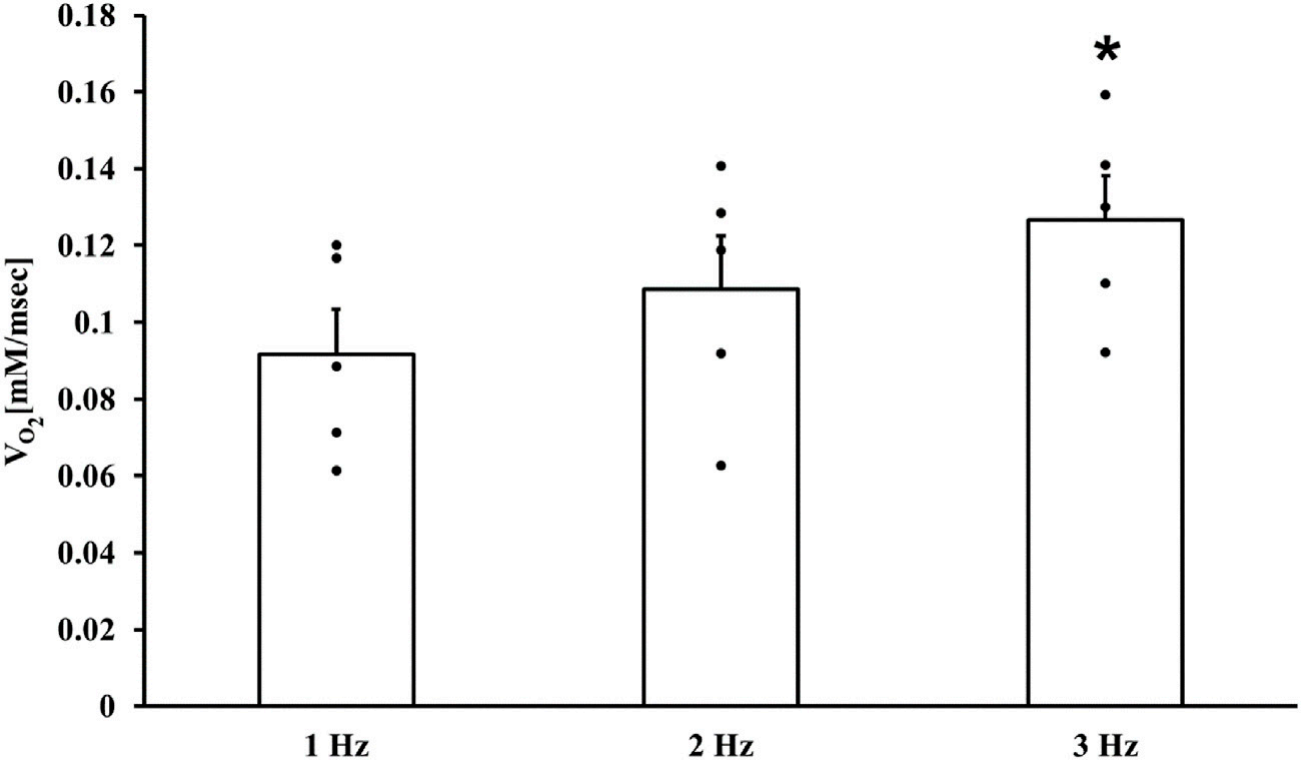
ATP supply in atrial cells. Respiration rates of isolated atrial cell suspensions (n = 5) electrically stimulated at 1, 2 and 3 Hz *p < 0.05 vs. 1 Hz.

Fundamental experiments were first performed to verify that an increase in electrical stimulus frequency increases ATP supply. In a fully coupled system, oxygen consumption is an indicator of ATP production, i.e., supply. Increases in stimulus frequency increased oxygen consumption and were statistically significant at 3 Hz compared to 1 Hz (Figure 2). Only the values of oxygen consumption at 1 Hz stimulus frequency were used as input to the computational model, and the predicted model values at 2 and 3 Hz stimulus frequency were compared to the experimental results (see below).

**FIGURE 3:**
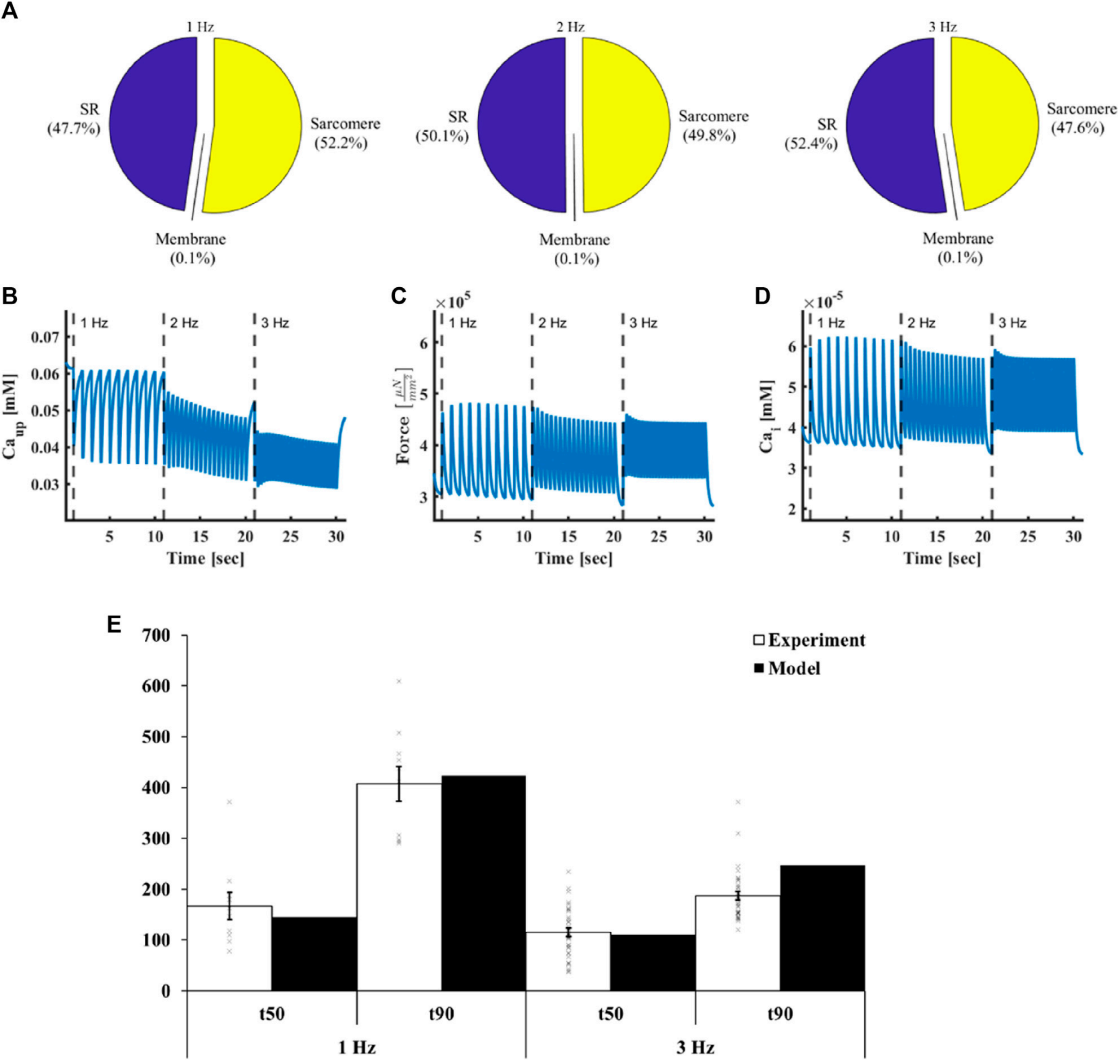
ATP demand in atrial cells. **(A)** Relative ATP consumption by sarcoplasmic reticulum Ca^2+^ cycling proteins, myofilaments and membranal pumps on exposure to electrical stimulation at different frequencies. **(B)** Ca^2+^ uptake by the sarcoplasmic reticulum, **(C)** force and **(D)** intracellular Ca^2+^ in response to electrical stimulus at frequencies 1, 2 and 3 Hz. **(E)** t_50_ (50% decline time of the Ca^2+^ signal from the peak) and t_90_ (90% decline time of the Ca^2+^ signal from the peak) achieved at 1 Hz and 3 Hz electrical stimulus frequencies both in the experiments (n = 36) and the model.

Using the computational model, we found that at 1 Hz st0069mulus frequency (Figure 3A), the SERCA pump (56.9%) and the myofilaments (43%) were the major ATP consumers, while maintenance of the membrane homeostasis (sodium-potassium pump and Ca^2+^ pump) consumed only 0.05% of the ATP budget. By calculating the relative consumption of ATP by the above consumers we found that increasing the stimulus frequency to 2 Hz and 3 Hz increased the ATP consumption rate by the SERCA and sarcomeres, due to an increased uptake rate (Figure 3B, from 3.48 pA at 1 Hz to 3.81 pA and 4.3 pA at 2 Hz and 3 Hz, respectively), and in the case of force, increased average systolic force (Figure 3C, from $3.31\cdot 10^{5}\frac{\mu N}{mm^{2}}$ at 1 Hz to $3.34\cdot 10^{5}\frac{\mu N}{mm^{2}}$ and $3.53\cdot 10^{5}\frac{\mu N}{mm^{2}}$ at 2 and 3 Hz, respectively). Supplementary Figure S1 shows the ionic currents and pumps measured in response to increasing electrical stimulus rates. The model predicted that the increase in ATP demand was mainly due to an increase in the rate, and not amplitude, of the currents because the amplitude of the sodium-potassium pump and Ca^2+^ pump currents slightly decreased with increased stimulus frequency. On change of the stimulus frequency (2 Hz and 3 Hz), there was no change in the relative ATP consumption by the consumers compared to 1 Hz. Thus, the rate-dependent changes in contractility and Ca^2+^ handling are the same, likely because the majority of Ca^2+^ is bound to troponin.

### 3.2 Ca^2+^ is an important regulator of ATP supply-to-demand matching

Our hypothesis was that Ca^2+^ (Figure 3D) is a key regulator of ATP supply-to-demand matching in the atrium. Thus, we experimentally measured its kinetic under different electrical stimulation rates. We found that at 1 Hz, the t_50_ (50% decline time of the Ca^2+^ signal from the peak) was 166.8 ± 168 ms and t_90_ (90% decline time of the Ca^2+^ signal from the peak) was 407 ± 204 ms (n = 36 cells). At 3 Hz, t_50_ was 115 ± 48 ms and t_90_ was 187 ± 48 ms, n = 36 (Figure 3E). We then fitted the parameters of the model, including channel and transporter dynamics and conductance, enzyme kinetics and initial values of state variables, to achieve similar Ca^2+^ kinetics at a 1 Hz stimulus frequency. The parameters were selected to fulfill additional criteria, which included ensuring model non-divergence, TCA cycle stability, and maintaining all state variables within physiological ranges. At 1 Hz stimulus frequency, the model predicted a t_50_ of 145.2 ms and t_90_ of 423.2 ms, which were both in the range of the experimental values (Figure 3E). We compared the model predictions to the experimental measurements at 3 Hz. At 3 Hz stimulus frequency, the model predicted a t_50_ of 110.2 ms and t_90_ of 247.1 ms, which aligned with the experimental results. Note that small differences in t_90_ may be related to frequency-dependent influx and outflux kinetics of Ca^2+^ from the SR.

**FIGURE 4:**
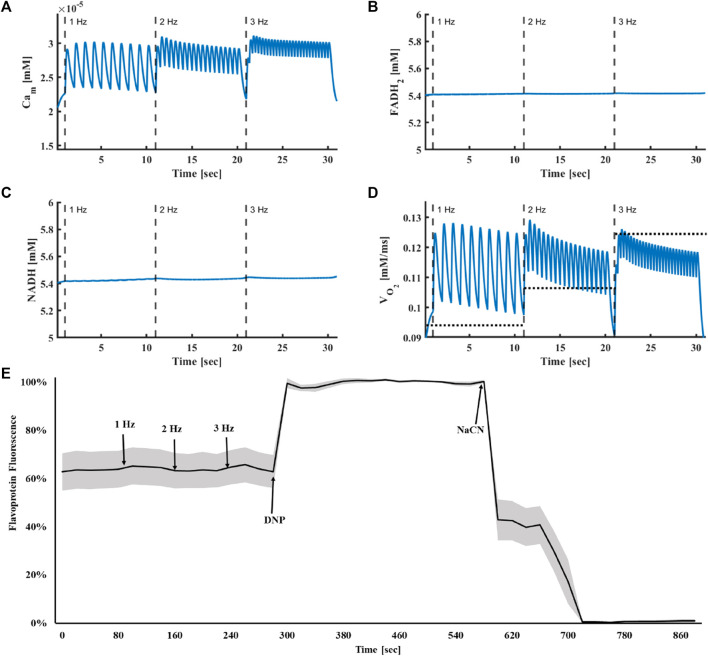
Metabolic homeostasis is response to electrical stimulus. **(A)** Mitochondrial Ca^2+^, **(B)** FADH_2_ and **(C)** NADH concentrations and **(D)** oxygen consumption in response to electrical stimulus at frequencies of 1, 2 and 3 Hz. The average experimental results are shown by the dashed line. **(E)** Experimental results of flavoprotein fluorescence in isolated atrial cells in response to electrical stimulus at frequencies of 1, 2 and 3 Hz (n = 7). To quantify the dynamic range of the oxidized vs. reduced flavoprotein pool, 100 μmol/L 2,4-dinitrophenol (DNP); (maximum flavoprotein fluorescence) and 4 mmol/L NaCN (reduced flavoproteins; minimal fluorescence) were added at the end of the experiment. The results are presented as mean (black line) with standard error (gray area).

**FIGURE 5:**
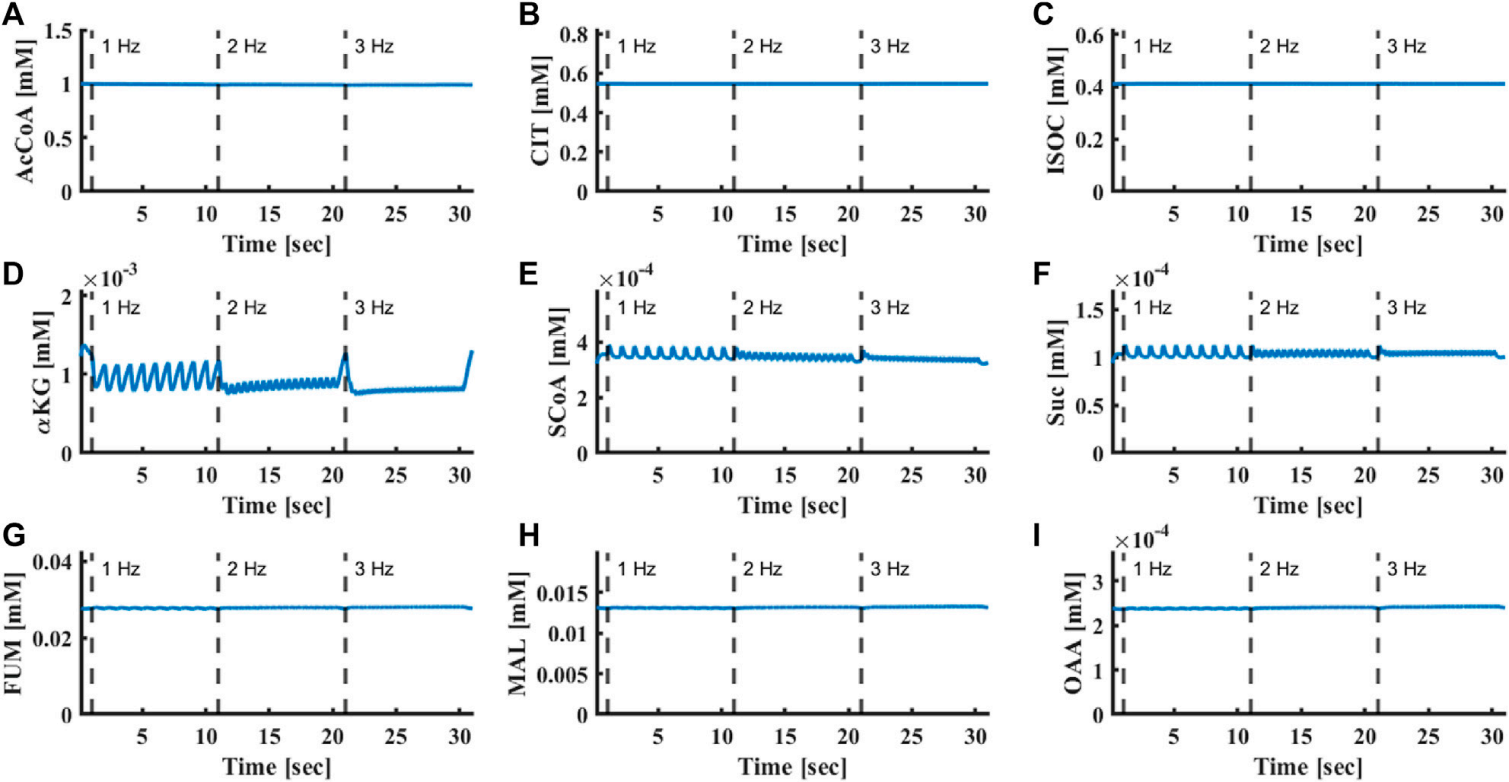
Oscillation of Krebs cycle protein concentrations. **(A)** Acetyl-CoA, **(B)** citrate, **(C)** isocitrate, **(D)** α-ketoglutarate, **(E)** succinyl-CoA, **(F)** succinate, **(G)** fumarate, **(H)** L-malate and **(I)** oxaloacetate concentrations in response to electrical stimulus at frequencies of 1, 2 and 3 Hz.

In the computational model, the increase in intracellular Ca^2+^ in response to an increase in stimulus frequency led to an accumulation of mitochondrial Ca^2+^ (Figure 4A, from $2.42\cdot 10^{-5}mM$ at 1 Hz to $2.63\cdot 10^{-5}mM$ and $2.81\cdot 10^{-5}mM$ at 2 and 3 Hz, respectively). With increased stimulation rates, metabolic homeostasis, quantified by FADH_2_ (Figure 4B, from 5.41 mM at 1 Hz to 5.41 mM and 5.42 mM at 2 and 3 Hz, respectively) or NADH (Figure 4C, from 5.42 mM at 1 Hz to 5.43 mM and 5.44 mM at 2 and 3 Hz, respectively) concentration, was maintained, while oxygen consumption increased (Figure 4D, from $0.104\frac{mM}{ms}$ at 1 Hz to $0.109\frac{mM}{ms}$ and $0.113\frac{mM}{ms}$ at 2 and 3 Hz, respectively) in a fashion similar to that measured experimentally (Figure 2). FADH_2_ levels remained constant upon increase of stimulus frequency from 1 Hz to 3 Hz, in accordance with previous experimental results (Kirschner Peretz et al., 2017) (Figure 4E). Similarly, the computational model predicted that metabolic homeostasis of all Krebs cycle metabolites was maintained (Figure 5). Note that although metabolic homeostasis existed in steady state, effects of Ca^2+^ oscillation on alpha ketoglutarate, succinyl-CoA and succinate were observed (Figures 5D–F). Yet, the oscillation amplitude was below the experimental detection level.

### 3.3 Ca^2+^ regulates ATP supply via both push and pull modes

**FIGURE 6:**
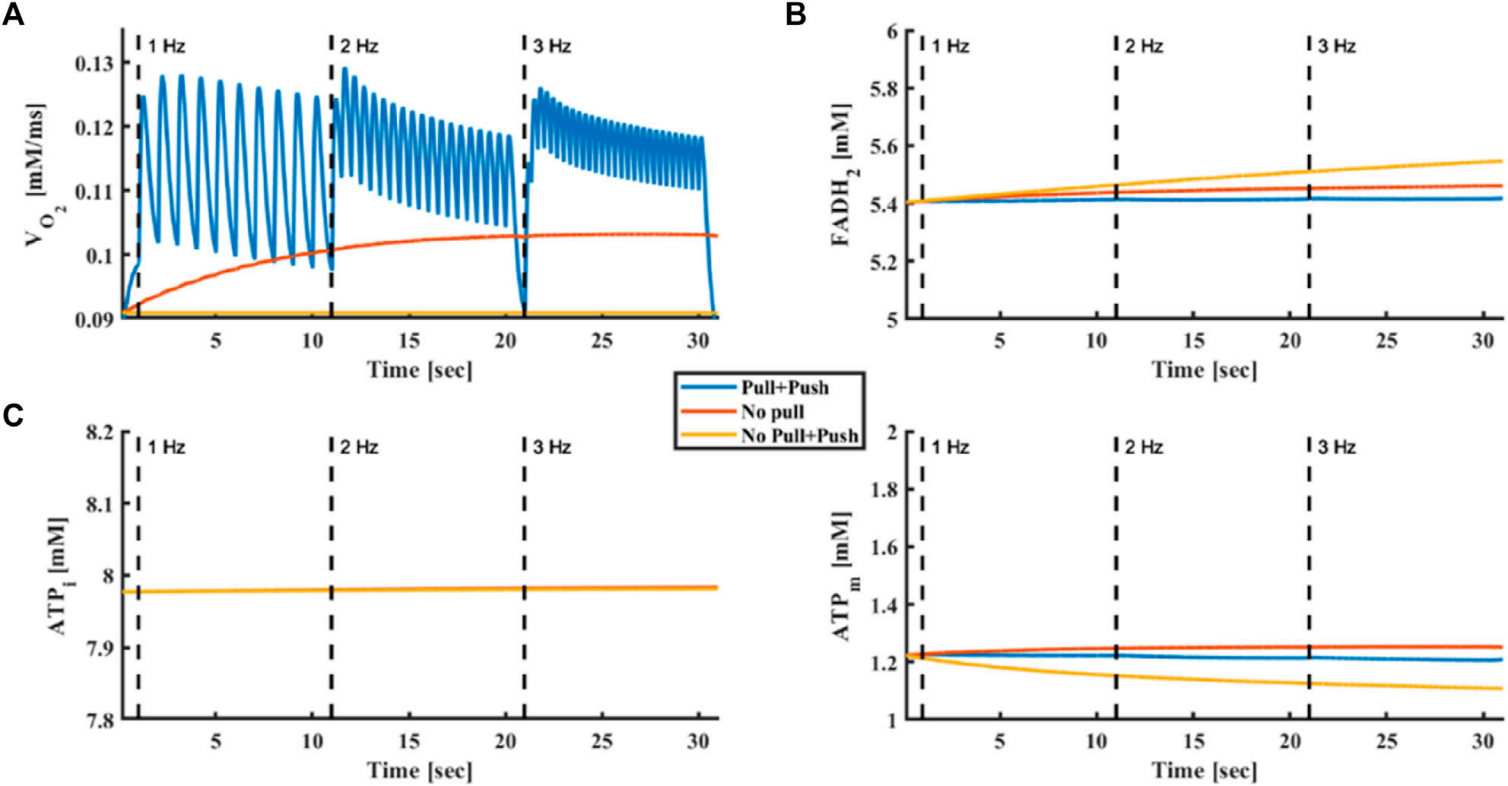
Push and pull mechanisms driven by Ca^2+^. **(A)** Oxygen consumption, **(B)** FADH_2_ concentration, **(C)** intracellular ATP and **(D)** mitochondrial ATP in response to electrical stimulus at frequencies of 1, 2 and 3 Hz. The model was tested without push and pull mechanisms driven by Ca^2+^, with only a Ca^2+^ push mechanism and with both push and pull mechanisms driven by Ca^2+^.

Next, we explored the regulation of ATP synthesis by Ca^2+^. Both push (mitochondrial Ca^2+^ activates metabolite flux and enzymes in the Krebs cycle) and pull (mitochondrial Ca^2+^ activates ATP synthase) mechanisms were suggested (Harris and Das, 1991). Figure 6 shows the simulated oxygen consumption (Figure 6A), FADH_2_ concentration (Figure 6B), intracellular (Figure 6C) and mitochondrial (Figure 6D) ATP levels in response to increasing stimulus frequencies. When both Ca^2+^ push and pull mechanisms were intact, oxygen consumption increased (from $0.104\frac{mM}{ms}$ at 1 Hz to $0.109\frac{mM}{ms}$ and $0.113\frac{mM}{ms}$ at 2 Hz and 3 Hz, respectively) in response to increased stimulus frequency, matching the experimental results, while metabolic homeostasis was maintained, as described elsewhere (Kirschner Peretz et al., 2017) and shown here (Figure 4E). However, when the ATP synthase pull mechanism was disabled prior to increased stimulus frequency, oxygen consumption did not change after reaching steady state (from $0.096\frac{mM}{ms}$ at 1 Hz to $0.101\frac{mM}{ms}$ and $0.103\frac{mM}{ms}$ at 2 and 3 Hz, respectively), excluding a transient effect at the beginning of the simulation, in contrast to experimental results. When both push and pull mechanisms were eliminated, no change in oxygen consumption was obtained (from $0.091\frac{mM}{ms}$ at 1 Hz to $0.091\frac{mM}{ms}$ and $0.091\frac{mM}{ms}$ at 2 Hz and 3 Hz, respectively), while mitochondrial ATP concentrations decreased and cellular ATP remained constant. Thus, in our simulation, the push mechanism was necessary to maintain metabolic homeostasis.

### 3.4 Metabolic homeostasis is maintained in response to change in stimulus frequency

**FIGURE 7:**
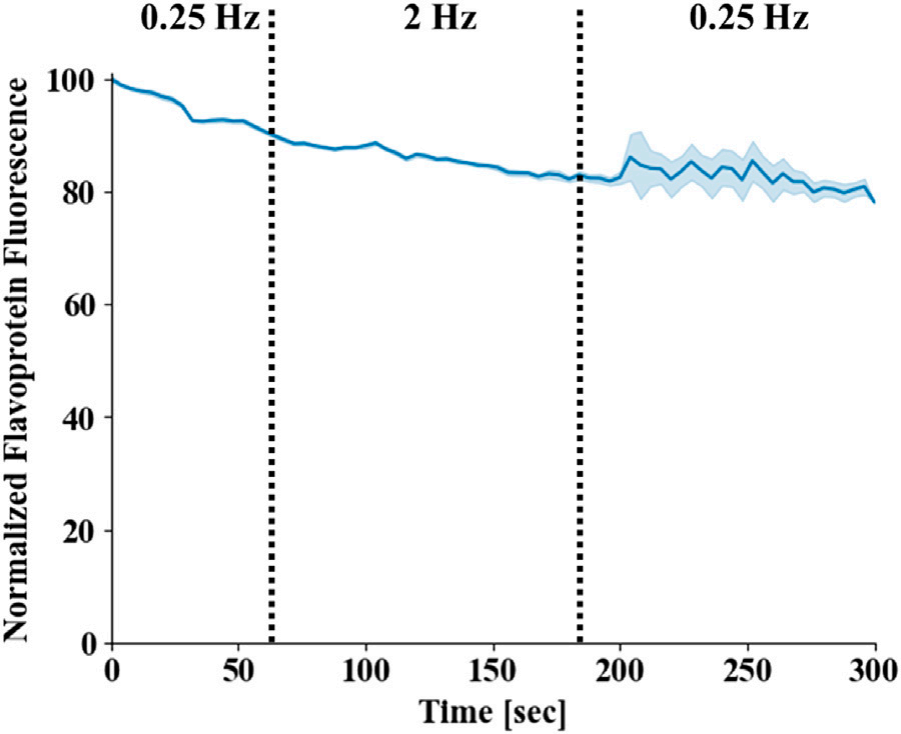
Flavoprotein homeostasis in atrial cells. Flavoprotein fluorescence in isolated atrial cells. The stimulus frequency was raised from 0.25 Hz to 2 Hz and then returned to 0.25 Hz (n = 4). Mean fluorescence is presented by a blue line and standard error by the light blue area around it. Note that the standard error increased towards the end of protocol, as the measurement of two of the cells was terminated before the end of the protocol due to technical issues.

Previous works have used computational models to demonstrate the existence of “push” and “pull” mechanisms in ventricular cells, which explain the time-dependent behavior of NADH (Cortassa et al., 2006). Others have measured the time-dependent behavior of NADH in ventricular rat trabeculae using a protocol in which the stimulus frequency was increased from 0.25 Hz to 2 Hz and then returned to 0.25 Hz (Brandes and Bers, 1999; 2002). We reproduced these experiment with a similar protocol in rabbit atrial cells. (Figure 7). When the frequency was increased from 0.25 Hz to 2 Hz (96.8% ± 5.6%) or when stimulus frequency was returned to 0.25 Hz after 2 Hz stimulation (95.2% ± 4.2) (n = 5 cells), no measurable frequency-dependent change was observed in flavoprotein fluorescence (a reciprocal proxy of NAD).

**FIGURE 8:**
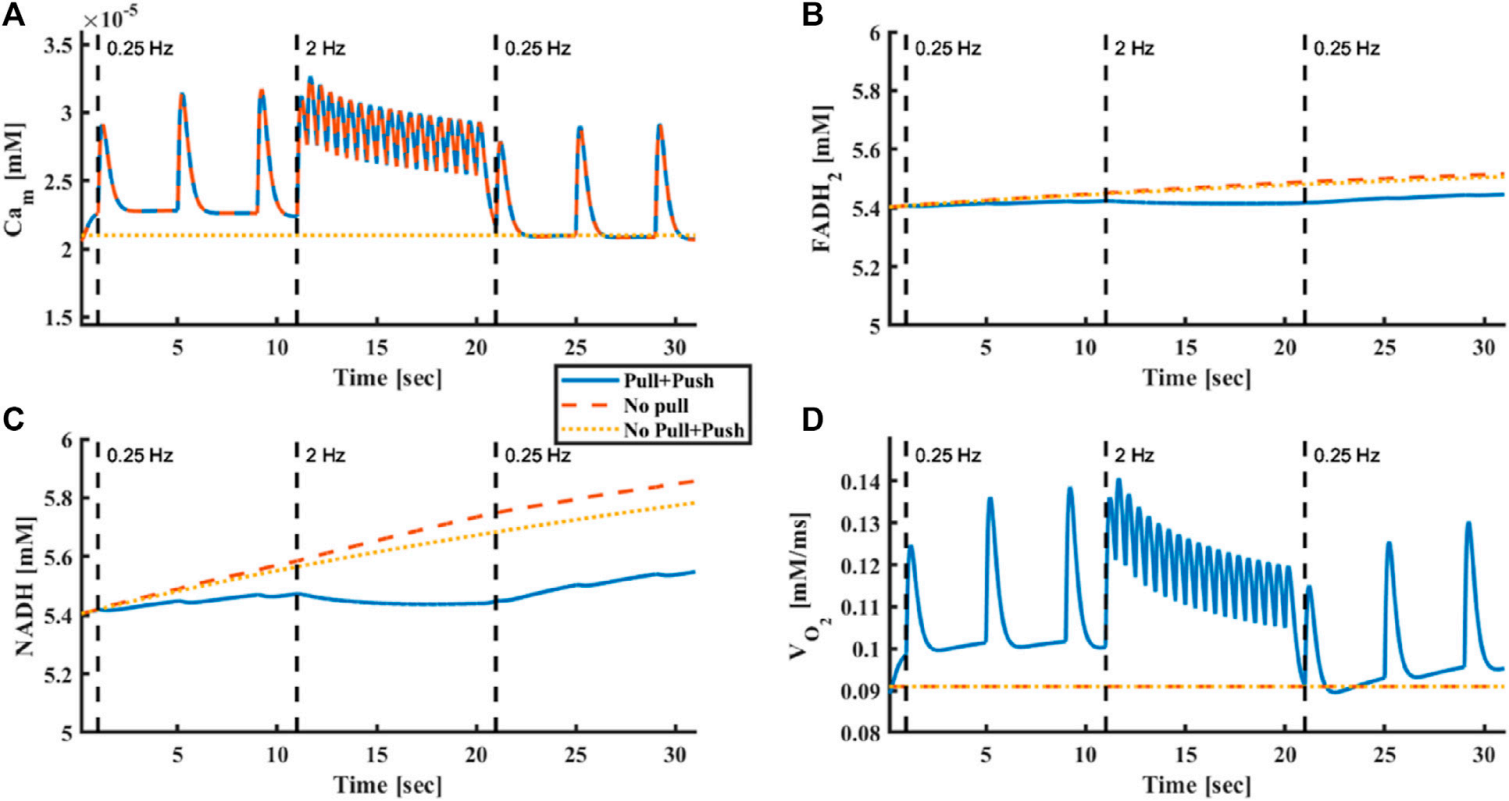
Model prediction of metabolic homeostasis is response to electrical stimulus. **(A)** Mitochondrial Ca^2+^, **(B)** FADH_2_ concentration, **(C)** NADH concentration and **(D)** oxygen consumption in response to electrical stimulus frequency that was raised from 0.25 Hz to 2 Hz and then returned to 0.25 Hz. The model was tested without Ca^2+^ push and pull mechanisms, with only Ca^2+^ push mechanism and with both Ca^2+^ push and pull mechanisms.

Our computational results showed that when the stimulus frequency was rapidly raised from 0.25 Hz to 2 Hz and then returned to 0.25 Hz, mitochondrial Ca^2+^ (Figure 8A from $2.38\cdot 10^{-5}mM$ at 0.25 Hz to $2.67\cdot 10^{-5}mM$ at 2 Hz), oxygen consumption (Figure 8D, from $0.105\frac{mM}{ms}$ at 0.25 Hz to $0.113\frac{mM}{ms}$ at 2 Hz) increased and decreased, respectively, but FADH_2_ (Figure 8C, from 5.414 mM at 0.25 Hz to 5.417 mM at 2 Hz) and NADH (Figure 8D, from 5.443 mM at 0.25 Hz to 5.446 mM at 2 Hz) levels were maintained. Note, that at the 2 Hz stimulation rate, a negative staircase of diastolic Ca^2+^ was observed. This phenomenon was likely due to changes in the kinetics of Ca^2+^ uptake and release from the SR. This phenomenon was consistent in all Ca^2+^ compartments. Nevertheless, the behavior of Ca^2+^ on all components across frequencies was as expected; higher frequencies elevated the mean cytoplasmic and mitochondrial Ca^2+^ concentrations, creating a higher contraction force in the sarcomeres, which signaled the mitochondria that energy consumption increased.

To confirm that Ca^2+^ plays a vital role in maintaining metabolic homeostasis during rapid increases in ATP demand, we disabled Ca^2+^ activation in either the push or pull pathways. With the pull mechanism disabled, no change in oxygen consumption occurred (from $0.096\frac{mM}{ms}$ at 0.25 Hz to $0.102\frac{mM}{ms}$ at 2 Hz), but metabolic homeostasis existed. However, when Ca^2+^ was absent from both the “pull” and “push” pathways, metabolic homeostasis was disrupted.

## 4 Discussion

In the present study, we investigated the role of Ca^2+^ in ATP supply-to-demand matching in atrial cells. We presented here, for the first time, a computational model that included both the main ATP consumers, mitochondrial bioenergetics and their regulators in atrial cells. The model predicted that increases in the electrical stimulation frequency increase ATP demand and mitochondrial Ca^2+^ in parallel to increased oxygen consumption. Clamping of mitochondrial Ca^2+^ resulted in the elimination of the coupling between stimulation rate and oxygen consumption, supporting the first hypothesis that Ca^2+^ is an important regulator of ATP supply-to-demand matching. In contrast to the experimental results, the model predicted that on elimination of the role of Ca^2+^ as a “pull” modulator, oxygen consumption does not increase in response to electrical stimulation, supporting the second hypothesis that Ca^2+^ acts both in “pull” and “push” modules. Finally, both experimental evidence and model predictions showed no rapid time-dependent changes in mitochondrial flavoprotein fluorescence in response to abrupt or slow changes in workload, supporting the third hypothesis that metabolic homeostasis is maintained in atrial cells in response to abrupt or slow increases in workload.

Our model predicted that Ca^2+^ is a key regulator of ATP supply-to-demand matching. At an increased workload, changes in mitochondrial Ca^2+^ were causally correlated with changes in oxygen consumption, while the other metabolites remained constant (i.e., showed no correlation with oxygen consumption). The comparison was enabled by novel oxygen consumption measurements, performed for the first time in isolated and electrically stimulated atrial cells. Ca^2+^ was regulated by both ATP supply and by the mitochondria that produce ATP. We simulated the two main ATP-consuming processes, both of which are Ca^2+^-dependent, i.e., pumping Ca^2+^ by the SERCA and force generation by myofilaments, to which the majority of Ca^2+^ is attached (Bers, 2002). Ca^2+^ enters the mitochondria through the mitochondrial Ca^2+^ uniporter (Baughman et al., 2011; De Stefani et al., 2011) and activates energy-related mechanisms, and is extruded by the mitochondrial Na^+^-Ca^2+^ exchanger (Palty et al., 2010). Eliminating the Ca^2+^ effect on ATP synthase (“pull” effect) diminished the correlation between mitochondrial Ca^2+^ and oxygen consumption. Moreover, without Ca^2+^ signaling, the metabolic homeostasis that we observed experimentally could not be maintained in the simulation. Thus, our results and simulation indicate that within the simulated workload range, Ca^2+^ is an important regulator of ATP supply to demand in both “push” and “pull” modes in atrial cells, as has been previously shown in ventricular cells (Cortassa et al., 2006; Bertero and Maack, 2018) and in isolated cardiac mitochondria (Banienë and Mildažienė, 2005). Note that when both the “push” and “pull” effects of Ca^2+^ were eliminated, ATP levels in the cytosol were still maintained in response to increases in workload, because presumably small changes in mitochondrial ADP pull the ATP synthase to increase ATP production. Thus, ADP is also an energy regulator in atrial cells.

It was postulated that “push” and “pull” modules lead to temporal changes in NADH levels in response to changes in workload (Cortassa et al., 2006). The differences in the relative response times of these two processes account for the transient overshoot and undershoot behavior of mitochondrial NADH documented in ventricular trabeculae (Brandes and Bers, 2002). However, in the current work, while Ca^2+^ “push” and “pull” effects were demonstrated in atrial cells, no transient overshoot or undershoot behavior was measured in response to change in overload. It is possible that different control mechanisms exist in atrial cells compared to the ventricle. Moreover, the experiments in ventricular trabeculae (Brandes and Bers, 2002) were performed at 22 °C, while the current work was conducted at 36 °C, which can result in different kinetics. Furthermore, it has been suggested that “push” is a Ca^2+^-dependent mechanism while “pull” is only an ATP-related and not Ca^2+^-dependent mechanism (Cortassa et al., 2006). It should be noted that Balaban and co-workers did not observe NADH transients in response to increased workload in the intact heart (Balaban, 2002) or in isolated mitochondria (Territo et al., 2001). Both here and in the isolated heart, the maintained metabolic homeostasis can be explained by the rapid increase in energy supply to meet energetic demands, triggered by fast parallel activation of the respiratory chain and Krebs cycle dehydrogenases by a cytoplasmic messenger, postulated here to be Ca^2+^. Note that based on the model, the changes in mitochondrial Ca^2+^ led to small changes (less than 2%) in the rate of tangible Ca^2+^-dependent effects on the Krebs cycle.

Our model faithfully reproduced experimental results. The model which was fitted on Ca^2+^ data measured under a 1 Hz stimulation rate, reproduced Ca^2+^ kinetics and oxygen consumption under a 3 Hz stimulation rate. The model also reproduced FADH_2_ homeostasis and predicted that metabolic homeostasis existed at all levels of bioenergetics, from the Krebs cycle enzymes to respiratory fluxes (Supplementary Figure S1). Note that all of our experiments were performed on rabbit cells, which exhibit excitation-contraction coupling mechanisms more similar to those of humans than mice, rats or guinea pigs (Bers, 2002). Although there are several promising computational models linking electrophysiology, ion homeostasis, Ca^2+^ handling, ATP consumption and mitochondrial energetics (Matsuoka et al., 2004; Cortassa et al., 2006; Yaniv et al., 2008), none of the models have been adjusted to atrial cells.

The model was developed to only consider supply-to-demand matching mechanisms working in a short window. It was shown before that several control mechanisms may work on a longer range (tens of seconds to minutes), such as CaMKII signaling (Yang et al., 2018) and the accumulation of intracellular sodium, which drive changes in Ca^2+^ handling via the sodium-calcium exchanger (Verkhratsky et al., 2018). Of note, even for short periods, there timing of activation of different mechanisms can differ; Ca^2+^ works on a beat-to-beat basis and was shown before to react faster than ADP to changes in demand (Cortassa et al., 2006).

## 5 Clinical insights

While a tight link between atrial fibrillation (AF) and electrophysiological and structural remodeling of the atria has been described (Voigt et al., 2014), recent evidence suggests that perturbations in energetic metabolites, which are tightly coupled with ion channel function and energy production, may be associated with transient or permanent AF. Alterations in Ca^2+^ handling have been correlated with AF (Dobrev and Nattel, 2008; Dobrev et al., 2011; Mason et al., 2020) and can lead to ATP supply-to-demand mismatch. Future experiments will be needed to retrain our model to provide predictions under AF conditions.

## 6 Limitations

The present model demonstrated the feasibility of mediation of energy supply-to-demand matching via Ca^2+^ and ADP in both “push” and “pull” modes. However, this work did not rule-out the possibility that other mechanisms and/or mediators are involved, such as posttranslational modification signaling, like PKA (Covian and Balaban, 2012). Additional experimental data on these signaling pathways will enable assessment of their relative role in ATP supply-to-demand matching.

Although cytosolic Ca^2+^ was measured in atrial cells, mitochondrial Ca^2+^ kinetics was not quantified. Such data are lacking due to the challenges of measurement of Ca^2+^ kinetics under high electrical stimulation frequencies. New methods are needed to complete this task.

The present work assumes that creatine kinase (CK) concentration is constant and does not model the CK kinetics in two compartments as was done in (Cortassa et al., 2006). In mice, near-total knockout of mitochondrial and/or cytosolic CK isoforms had a modest but measurable effect on cardiac performance measurements under normal as well as increased-demand conditions (Lygate et al., 2009; Lygate et al., 2013). Thus, under normal conditions the role of CK in ATP production is negligible. Future experiments will be necessary to understand the importance of the CK shuttle in ATP supply-to-demand matching in the atria.

Our computational model represented only the control mechanisms that operate on a time scale of seconds. Electrical stimulation for several minutes may activate additional energy balance maintenance mechanisms. Of note, even for longer periods of electrical stimulation (1 minute) metabolic homeostasis was maintained experimentally (Kirschner Peretz et al., 2017).

One should also take into consideration that some control mechanisms, such as Ca^2+^, work on a beat-to-beat basis. However, the tools we used to quantify bioenergetics do not support such a level of resolution.

## 7 Conclusion

This work measured oxygen consumption and metabolic balance indicators in rabbit atrial cells and developed an integrated model of the electrophysiology, excitation-contraction coupling and mitochondrial bioenergetics of atrial cells. The model reproduced the increase in oxygen consumption in response to electrical stimulation and maintained metabolic homeostasis under abrupt changes in workload. The model predicted that Ca^2+^ is an important regulator of ATP supply-to-demand matching and that Ca^2+^ works in both “push” and “pull” directions.

## Data Availability

The original contributions presented in the study are included in the article/Supplementary Material, further inquiries can be directed to the corresponding author.

## Abbreviation

AF, Atrial fibrillation; ATP, Adenosine triphosphate; Ca^2+^m, Mitochondrial calcium; CK, Creatine kinase; PDH, Pyruvate dehydrogenase; SR, Sarcoplasmic reticulum.
