# Supplementary material for: Ca2+ pushes and pulls energetics to maintain ATP balance in atrial cells: computational insights
Source: Front Physiol. 2023 Jul 17;14:1231259. doi: 10.3389/fphys.2023.1231259 (PMC10387757; doi:10.3389/fphys.2023.1231259)
Supplement: Supplementary file 1 [file DataSheet1.docx]

**Ca^2+^ pushes and pulls energetics to maintain ATP balance in atrial cells: computational insights**

**Supplemental material**

Our computational model integrates several specific models, each representing a compartment or phenomenon in the cell, to jointly include all the feedback and control loops that govern the phenomena focused on in this research.

1. The sub-models include: membranal ionic currents, pumps and exchangers
2. Sarcoplasmic reticulum (SR) Ca2^+^ handling
3. Force generation and sarcomere energy consumption
4. Mitochondrial energy metabolism
5. Mitochondrial Ca^2+^ dynamics

**a.** **Membranal ionic currents, pumps and exchangers**

This part of the model is based on the rabbit atrial cell model described by Lindblad et al. (1), which is presented here for the readers’ convenience. Their model consists of two components: an equivalent electrical circuit corresponding to the sarcolemmal ionic channels, pumps and exchangers and a model of the intracellular space molecules and SR compartments, presented in the next section.

The sarcolemma is modeled as a capacitor (C_m_), powered by various ionic currents, pumps and exchangers, which have been previously shown to exist in the rabbit atrial cell membrane (2). We assumed that the transmembranal potential (V) is spatially uniform and time varying, and that the total net ionic current ($I_{net}$) is linearly related to the time derivative of V:

1. $I_{\mathrm{net}}=\sum I_{\mathrm{ionic}}=-C_{m}\frac{\mathrm{dV}}{\mathrm{dt}}$.

This model utilizes Hodgkin-Huxley (HH) style equations for each ionic current using the following general equation:

1. $I_{i}=\bar{g_{i}}{y_{i,1}}^{k_{1}}\cdot\cdot\cdot\cdot\cdot{y_{i,n}}^{k_{n}}(V-E_{i})$,

where E_i_ is the reversal potential for the i^th^ ionic species, $\bar{g_{i}}$ is the maximal conductance for the i^th^ current, and the $y_{i,j}$ terms are HH-type gating variables with the exponents $k_{i}$ for the i^th^ ionic current characteristics.

The gating variables, through HH equation characteristics, follow first-order kinetics through the general equation:

1. $\frac{\mathrm{dy}}{\mathrm{dt}}\left( V,t \right)=\frac{y_{\infty}\left( V \right)-y(V,t)}{\tau_{y}(V)}$,

where $y_{\infty}$ represents the steady state value of y as a function of V and $\tau_{y}$ is the voltage-dependent time constant of y.

To summarize, this part of our model assumes that: (i) the extracellular ionic concentrations are constant, (ii) the cell model is a single-component model for the distribution of Na^+^ and K^+^, (iii) the model equations are based on HH formalism, and (iv) the atrial cell possesses a uniform cylindrical geometry.

The reversal (“Nernst”) potentials of the main ions are calculated by:

1. $E_{k}=\frac{\mathrm{RT}}{F}\ln\left( \frac{\left[ K^{+} \right]_{o}}{\left[ K^{+} \right]_{i}} \right)$
2. $E_{\mathrm{Na}}=\frac{\mathrm{RT}}{F}\ln\left( \frac{\left[ Na^{+} \right]_{o}}{\left[ Na^{+} \right]_{i}} \right)$
3. $E_{\mathrm{Ca}}=\frac{\mathrm{RT}}{2F}\ln\left( \frac{\left[ Ca^{2+} \right]_{o}}{\left[ Ca^{2+} \right]_{i}} \right)$,

where$\left[ K^{+} \right]_{o} , \left[ Na^{+} \right]_{o} , \left[ Ca^{2+} \right]_{o}$and $\left[ K^{+} \right]_{i} , \left[ Na^{+} \right]_{i} , \left[ Ca^{2+} \right]_{i}$are the ion concentrations in the outside and in the cytoplasm space, respectively, R is the ideal gas constant, T is the temperature in Kelvin and F is Faraday’s constant.

The model parameters are summarized in Table S1 and the initial values of the state variables are presented in Table S2.

*i. Fast-delayed rectifier* $K^{+}$ *current*

1. $I_{k,r}=g_{K^{+},r}p_{a}p_{i}\left( V-E_{k} \right)$
2. $\frac{dp_{a}}{\mathrm{dt}}=\frac{p_{a,\infty}-p_{a}}{\tau_{p_{a}}}$
3. $p_{a,\infty}=\frac{1}{1+e^{-\left( \frac{V+5.1}{7.4} \right)}}$
4. $\tau_{p_{a}}=\frac{1}{\alpha_{p_{a}}+\beta_{p_{a}}}$
5. $\alpha_{p_{a}}=9\cdot e^{\frac{V}{25.371}}$
6. $\beta_{p_{a}}=1.3\cdot e^{-\frac{V}{13.026}}$
7. $\frac{dp_{i}}{\mathrm{dt}}=\frac{\left( p_{i,\infty}-p_{i} \right)}{\tau_{p_{i}}}$
8. $p_{i,\infty}=\frac{1}{1+e^{\frac{V+47.3921}{18.6603}}}$
9. $\tau_{p_{i}}=\frac{1}{\alpha_{p_{i}}+\beta_{p_{i}}}$
10. $\alpha_{p_{i}}=100\cdot e^{-\frac{V}{54.645}}$
11. $\beta_{p_{i}}=656\cdot e^{\frac{V}{106.157}}$,

where $g_{K^{+},r}$ is the maximal conductance for the fast-delayed rectifying $K^{+}$ channels.

*ii. Slow-delayed rectifier* $K^{+}$ *current*

1. $I_{K^{+},s}=g_{K^{+},s}n\left( V-E_{K} \right)$
2. $\frac{\mathrm{dn}}{\mathrm{dt}}=\frac{n_{\infty}-n}{\tau_{n}}$
3. $n_{\infty}=\frac{1}{1+e^{-\frac{V-0.9}{13.8}}}$
4. $\alpha_{n}=1.66\cdot e^{\frac{V}{69.452}}$
5. $\beta_{n}=0.3\cdot e^{-\frac{V}{21.826}}$
6. $\tau_{n}=\frac{1}{\alpha_{n}+\beta_{n}}+0.06$,

where $g_{K^{+},s}$ is the maximal conductance for the slow-delayed rectifying $K^{+}$ channels.

*iii. Inward rectifier* $K^{+}$ *channel*

1. $I_{K1}=2.5\cdot g_{K1}\left( \frac{\left[ K^{+} \right]_{\mathrm{out}}}{\left[ K^{+} \right]_{\mathrm{out}}+k_{m,K1}} \right)^{3}\frac{V-E_{K}}{1+e^{1.393\left( V-E_{K}+3.6 \right)\cdot\frac{F}{\mathrm{RT}}}}$,

where$g_{K1}$ is the maximal conductance of the Inward rectifier $K^{+}$ channels and $k_{m,K1}$ is the equilibrium binding constant for K^+^ dependence of the Inward rectifier $K^{+}$ channel channels.

*iv. Transient outward* $K^{+}$ *channel*

1. $I_{K,to}={0.35\cdot g}_{K,to}r\left( 0.59s_{1}^{3}+ 0.41s_{2}^{3} \right)\left( 0.6 s_{3}^{6}+0.4 \right)\left( V-E_{K} \right)$
2. $\frac{\mathrm{dr}}{\mathrm{dt}}=\frac{r_{\infty}-r}{\tau_{r}}$
3. $r_{\infty}=\frac{1}{1+e^{-\frac{V+15}{5.633}}}$
4. $\alpha_{r}=386.6\cdot e^{\frac{V}{12}}$
5. $\beta_{r}=8.011\cdot e^{-\frac{V}{7.2}}$
6. $\tau_{r}=\frac{1}{\alpha_{r}+\beta_{r}}+0.0004$
7. $\frac{ds_{1}}{\mathrm{dt}}=\frac{s_{1,\infty}-s_{1}}{\tau_{s_{1}}}$
8. $s_{1,\infty}=\frac{1}{1+e^{\frac{V+28.29}{7.06}}}$
9. $\tau_{s_{1}}=\frac{0.5466}{1+e^{\frac{V+32.8}{0.1}}}+0.0204$
10. $\frac{ds_{2}}{\mathrm{dt}}=\frac{s_{2,\infty}-s_{2}}{\tau_{s_{2}}}$
11. $s_{2,\infty}= \frac{1}{1+e^{\frac{V+28.29}{7.06}}}$
12. $\tau_{s_{2}}=\frac{5.75}{1+e^{\frac{V+32.8}{0.1}}}+\frac{0.45}{1+e^{-\frac{V-13.54}{13.97}}}$
13. $\frac{ds_{3}}{\mathrm{dt}}=\frac{s_{3,\infty}-s_{3}}{\tau_{s_{3}}}$
14. $s_{3,\infty}= \left( \frac{1}{1+e^{\frac{V+50.67}{27.38}}}+0.666 \right)/1.666$
15. $\tau_{s_{3}}=\frac{7.5}{1+e^{\frac{V+23}{0.5}}}+0.5$,

where $g_{K,to}$is the maximal conductance of the transient outward $K^{+}$ channels.

*v. Sustained outward current*

1. $I_{\mathrm{sus}}=g_{\mathrm{sus}}\left( V-E_{\mathrm{sus}} \right)$
2. $I_{K,to}=I_{K,to}+I_{\mathrm{sus}}$,

where $g_{sus}$is the maximal conductance of the sustained outward current and $E_{\mathrm{sus}}$ is the reversal potential of the sustained outward currents.

*vi. Na^+^-K^+^- ATPase*

1. $I_{\mathrm{NaK}}=I_{NaK,max}\left( \frac{\left[ K^{+} \right]_{\mathrm{out}}}{\left[ K^{+} \right]_{\mathrm{out}} + k_{m,K}} \right)\left( \frac{\left[ Na^{+} \right]_{\mathrm{in}}^{1.5}}{\left[ Na^{+} \right]_{\mathrm{in}}^{1.5} + k_{m,Na}} \right)\left( \frac{1.6}{1.5+e^{-\frac{V+60}{40}}} \right)$,

where $I_{NaK,max}$ is the maximal current of the Na-K pumps, $k_{m,K}$ is the equilibrium binding constant of K^+^ to the pump and $k_{m,Na}$ is the equilibrium binding constant of Na^+^.

*vii. Na^+^-Ca^2+^ exchanger (NCX)*

1. $I_{\mathrm{NaCa}}=\frac{0.02\left( \left[ \mathrm{Na} \right]_{\mathrm{in}}^{3}\left[ Ca^{2+} \right]_{\mathrm{out}}e^{\gamma_{\mathrm{NaCa}}\frac{\mathrm{VF}}{\mathrm{RT}}} -\left[ Na^{+} \right]_{\mathrm{out}}^{3}\left[ Ca^{2+} \right]_{i}e^{V\left( \gamma_{\mathrm{NaCa}}-1 \right)\frac{F}{\mathrm{RT}}} \right)}{1 + d_{\mathrm{NaCa}}\left( \left[ Ca^{2+} \right]_{i}\left[ Na^{+} \right]_{\mathrm{out}}^{3} +\left[ Ca^{2+} \right]_{\mathrm{out}}\left[ Na^{+} \right]_{\mathrm{in}}^{3} \right)}$,

where $\gamma_{\mathrm{NaCa}}$ is the position of Eyring rate theory energy barrier controlling voltage dependence of the NCX, and $d_{\mathrm{NaCa}}$ is a empirically fitted constant .

*viii. Fast Na^+^ channels*

1. $I_{\mathrm{Na}}=g_{\mathrm{Na}}m^{3}h\left[ Na^{+} \right]_{\mathrm{out}}\frac{VF^{2}}{\mathrm{RT}}\frac{e^{\left( V-E_{\mathrm{Na}} \right)\frac{F}{\mathrm{RT}}}-1}{e^{\frac{\mathrm{VF}}{\mathrm{RT}}}-1}$
2. $\frac{\mathrm{dm}}{\mathrm{dt}}=\alpha_{m}\left( 1-m \right)-\beta_{m}m$
3. $\alpha_{m}=-460\frac{V+44.4}{e^{-\frac{V+44.4}{12.673}}-1}$
4. $\beta_{m}=18400e^{-\frac{V+44.4}{12.673}}$
5. $h=0.635h_{1}+0.365h_{2}$
6. $\frac{dh_{1}}{\mathrm{dt}}=\frac{h_{\infty}-h_{1}}{\tau_{h_{1}}}$
7. $\frac{dh_{2}}{\mathrm{dt}}=\frac{h_{\infty}-h_{2}}{\tau_{h_{2}}}$
8. $h_{\infty}=\frac{\alpha_{h}}{\alpha_{h}+\beta_{h}}$
9. $\alpha_{h}=44.9e^{-\frac{V+66.9}{5.57}}$
10. $\beta_{h}=\frac{1491.0}{1+323.3e^{-\frac{V+94.6}{12.9}}}$
11. $\tau_{h_{1}}=\frac{0.03}{1 + e^{\frac{V+40}{6}}} + 0.00015$
12. $\tau_{h_{2}}=\frac{0.12}{1 + e^{\frac{V+60}{2}}} + 0.00045$ ,

|where $g_{\mathrm{Na}}$ is the maximal conductance of the fast sodium channels.

*ix. Membranal Ca^2+^ pump*

1. $I_{Ca,p} = I_{Cap,max}\left( \frac{\left[ Ca^{2+} \right]_{\mathrm{in}}}{\left[ Ca^{2+} \right]_{\mathrm{in}} + 0.0002} \right)$,

where $I_{Cap,max}$ is the maximal current of the membranal $Ca^{2+}$ pumps.

*x. L-type Ca^2+^ channels*

1. $I_{Ca,L}=g_{Ca,L}\left( d_{L}f_{L}+\frac{1}{1+e^{-\frac{V-23}{12}}} \right)\left( V-E_{Ca,L} \right)$
2. $\frac{dd_{L}}{\mathrm{dt}}=\frac{d_{L,\infty}-d_{L}}{\tau_{d_{L}}}$
3. $d_{L,\infty}=\frac{1}{1+e^{-\frac{V+10.95}{6.6}}}$
4. $\tau_{d_{L}}=\frac{1}{\alpha_{d_{L}}+\beta_{d_{L}}}$
5. $\alpha_{d_{L}}= -16.72 \frac{V + 45}{1-e^{-\frac{V+45}{2.5}}}+ 50.0\frac{V+10}{1-e^{-\frac{V+10}{4.808}}}$
6. $\beta_{d_{L}}=-4.48\frac{V+5}{1-e^{\frac{V+5}{2.5}}}$
7. $\frac{df_{L}}{\mathrm{dt}}=\frac{f_{L,\infty}-f_{L}}{\tau_{f_{L}}}$
8. $f_{L,\infty}=\frac{\alpha_{f_{L}}}{\alpha_{f_{L}}+\beta_{f_{L}}}$
9. $\tau_{f_{L}}=\frac{1}{\alpha_{f_{L}}+\beta_{f_{L}}}$
10. $\alpha_{f_{L}}=-2.1225\frac{V + 18}{1-e^{\frac{V + 18}{4}}}$
11. $\beta_{f_{L}}=\frac{67.922}{1 + e^{-\frac{V+18}{4}}}$,

where $g_{Ca,L}$ is the maximal conductance of the L-type calcium channels and $E_{Ca,L}$ is the reversal potential for the L-type calcium channels. Note that $E_{Ca,L}$ doesn’t necessary match the Nernst potential of Ca^2+^ in the cell.

*xi. T-type Ca^2+^ channels*

1. $I_{Ca,T}=g_{Ca,T}d_{T}f_{T}\left( V-E_{Ca,T} \right)$
2. $\frac{dd_{T}}{\mathrm{dt}}=\frac{d_{T,\infty}-d_{T}}{\tau_{d_{T}}}$
3. $d_{T,\infty}=\frac{1}{1 + e^{-\frac{\left( V + 23 \right)}{6.1}}}$
4. $\tau_{d_{T}}=\frac{1}{\alpha_{d_{T}}+\beta_{d_{T}}}$
5. $\alpha_{d_{T}}=674.173e^{\frac{V+23.3}{30}}$
6. $\beta_{d_{T}}=674.173e^{-\frac{V + 23.3}{30}}$
7. $\frac{df_{T}}{\mathrm{dt}}=\frac{f_{T,\infty}-f_{T}}{\tau_{f_{T}}}$
8. $f_{T,\infty}=\frac{\alpha_{f_{T}}}{\alpha_{f_{T}}+\beta_{f_{T}}}$
9. $\tau_{f_{t}}=\frac{1}{\alpha_{f_{T}}+\beta_{f_{T}}}$
10. $\alpha_{f_{T}}=9.637e^{-\frac{V+75}{83.3}}$
11. $\beta_{f_{T}}=9.637e^{\frac{V + 75}{15.38}}$,

where $g_{Ca,T}$ is the maximal conductance of the T-type calcium channels and $E_{Ca,T}$ is the reversal potential for the T-type calcium channels. Note that $E_{Ca,T}$ doesn’t necessarily match the Nernst potential of Ca^2+^ in the cell.

*xii. Background currents*

1. $I_{Na,b}=g_{Na,b}\left( V-E_{\mathrm{Na}} \right)$
2. $I_{Ca,b}=g_{Ca,b}\left( V-E_{\mathrm{Ca}} \right)$,

where $g_{Na,b}$ and $g_{Ca,b}$ are the leakage conductance for Na^+^ and Ca^2+^, respectively.

*xiii. Total membranal current*

1. $I_{\mathrm{tot}}=I_{K,r}+I_{K,s}+I_{K1}+I_{K,to}+I_{\mathrm{NaK}}+I_{\mathrm{NaCa}}+I_{\mathrm{Na}}+I_{Na,b}+I_{Ca,L}+I_{Ca,T}+I_{Ca,p}+I_{Ca,b}$

*xiv. Sodium and potassium concentrations in cytoplasm*

1. $\frac{d\left[ Na^{+} \right]_{\mathrm{in}}}{\mathrm{dt}}=\frac{-3I_{\mathrm{NaK}}-3I_{\mathrm{NaCa}}-I_{Na,b}-I_{\mathrm{Na}}}{V_{i}F}$
2. $\frac{d\left[ K^{+} \right]_{\mathrm{out}}}{\mathrm{dt}}=-\frac{2I_{\mathrm{NaK}}-I_{k,r}-I_{K,s}-I_{K1}-I_{K,to}}{V_{c}F}$
3. $\frac{d\left[ K^{+} \right]_{\mathrm{in}}}{\mathrm{dt}}=-\frac{-2I_{\mathrm{NaK}}+I_{k,r}+I_{K,s}+I_{K1}+I_{K,to}}{V_{i}F}$,

where $V_{i}$ is the intracellular volume and $V_{c}$ is the cytoplasmic volume.

**Table S1: Ionic current, exchanger and pump parameters**

| **Units** | **Value** | **Description** | **Symbol** |
| --- | --- | --- | --- |
| C/m | 96847 | Faraday's constant | F |
| mJ/(M·K˚) | 8314 | Gas constant | R |
| K° | 308 | Temperature | T |
| pF | 0.05 | Cell capacitance | C_m_ |
| mM | 2.5 | Extracellular Ca^2+^ | [Ca^2+^]_o_ |
| mM | 140 | Extracellular Na^+^ | [Na^+^]_o_ |
| mM | 5 | Extracellular K^+^ | [K^+^]_o_ |
| nL | 0.0126 | Cytoplasmic volume | V_i_ |
| nL | 0.005884 | Ca^2+^ intracellular volume | V_Ca_ |
| nL | 0.0025 | Cell volume | V_c_ |
| nL | 0.0003969 | SR uptake compartment volume | V_up_ |
| nL | 0.000044 | SR release compartment volume | V_rel_ |
| nS | 3.5 | Maximal conductance for fast-delayed rectifying K^+^ channels | g_K,r_ |
| nS | 2.5 | Maximal conductance for slow-delayed rectifying K^+^ channels | g_K,s_ |
| nS | 5.08 | Maximal conductance for inward rectifying K^+^ channels | g_K1_ |
| [] | 0.59 | Equilibrium binding constant for K^+^ dependence of the I_K1_ channels | k_mK1_ |
| nS | 500.2 | Maximal conductance for transient outward K^+^ channels | g_K,to_ |
| nS | 2.4 | Maximal conductance for sustained outward K^+^ channels | g_sus_ |
| pA | 64.41 | Maximal current of the Na^+^-K^+^ pumps | I_NaK,max_ |
| [] | 1 | Equilibrium binding constant of K^+^ to the pump | k_m,k_ |
| [] | 36.48 | Equilibrium binding constant of Na^+^ to the pump | k_m,Na_ |
| [] | 0.45 | Position of Eyring rate theory energy barrier controlling voltage dependence of NCX | γ_NaCa_ |
| [] | 0.0003 | Denominator constant for I_NaCa_ | d_NaCa_ |
| nS | 0.003 | Maximal conductance for Na^+^ channels | g_Na_ |
| pA | 9.509 | Maximal current of the membranal Ca^2+^ pumps | I_CaP,max_ |
| mV | 50 | Reversal potential of L-type Ca^2+^ channels | E_Ca,L_ |
| nS | 2.1 | Maximal conductance of L-type Ca^2+^ channels | g_Ca,L_ |
| mV | 38 | Reversal potential of T-type Ca^2+^ channels | E_Ca,T_ |
| nS | 60 | Maximal conductance of T-type Ca^2+^ channels | g_Ca,T_ |
| nS | 0.03 | Leakage conductance for Na | g_Na,b_ |
| nS | 0.03 | Leakage conductance for Ca | g_Ca,b_ |

**Table S2: Initial values of state variables**

| **Symbol** | **Description** | **Value** | **Units** |
| --- | --- | --- | --- |
| V | Transmembrane potential | -80 | mV |
| p_a_ | Activation gating variable for I_K,r_ | 4.0199∙10^-5^ | [] |
| p_i_ | Inactivation gating variable for I_K,r_ | 0.8516 | [] |
| N | Activation gating variable of I_K,s_ | 0.0283 | [] |
| R | Activation gating variable of $I_{K,to}$ | 9.7411∙10-6 | [] |
| s_1_ | Fast inactivation gating variable for $I_{K,to}$ | 0.9934 | [] |
| s_2_ | Slow inactivation gating variable for $I_{K,to}$ | 0.9934 | [] |
| s_3_ | Third inactivation gating variable for $I_{K,to}$ | 0.8468 | [] |
| M | Activation gating variable for I_Na_ | 0.01309 | [] |
| h_1_ | Fast inactivation gating variable for I_Na_ | 0.706 | [] |
| h_2_ | Slow inactivation gating variable for I_Na_ | 0.6149 | [] |
| d_L_ | Activation gating variable for I_Ca,L_ | 2.8599∙10-5 | [] |
| f_L_ | Inactivation gating variable for I_Ca,L_ | 1 | [] |
| d_T_ | Activation gating variable for I_Ca,T_ | 8.7458∙10-5 | [] |
| f_T_ | Inactivation gating variable for I_Ca,T_ | 0.5951 | [] |
| [Na]_i_ | Intracellular Na+ concentration | 8.4 | mM |
| [Ca^2+^]_up_ | Ca^2+^ concentration in uptake compartment | 0.069 | mM |
| [Ca^2+^]_rel_ | Ca^2+^ concentration in release compartment | 0.08 | mM |
| [Ca^2+^]_i_ | Ca^2+^ concentration in intracellular medium | 0.0000243 | mM |
| O_c_ | Fractional occupancy of calmodulin by Ca^2+^ | 0.029108 | [] |
| O_TnCa_ | Fractional occupancy of troponin-Ca complex by Ca^2+^ | 0.01407 | [] |
| O_TnMgCa_ | Fractional occupancy of troponin-Mg complex by Ca^2+^ | 0.214 | [] |
| O_TnMgMg_ | Fractional occupancy of troponin-Mg complex by Mg^2+^ | 0.6936 | [] |
| O_calse_ | Fractional occupancy of calsequestrin by Ca^2+^ | 0.09674 | [] |
| Ko | Extracellular K^+^ concentration | 5 | mM |
| K_i_ | Intracellular K^+^ concentration | 140 | mM |
| $F_{1}$ | Relative amount of inactive precursor in SR release compartment | 0.288 | [] |
| $F_{2}$ | Relative amount of activator in SR release compartment | 0.002262 | [] |
| $F_{3}$ | Relative amount of inactive product in SR release compartment | 0.612697 | [] |
| SL | Sarcomere length | 1.75 | µm |
| A | The density of regulatory units with bound Ca^2+^ and adjacent weak cross-bridges | 0.0233 | [] |
| TT | The density of regulatory units with bound Ca^2+^ and adjacent strong cross-bridge | 0.0018 | [] |
| U | The density of regulatory units without bound but with adjacent strong cross-bridge | 0.0293 | [] |
| V_e_ | Velocity | 0 | µm/sec |
| [ATP]_i_ | EC-coupling-linked ATP concentration | 7.972 | mM |
| [Ca^2+^]_m_ | Mitochondrial free Ca^2+^ concentration | 0.000021 | mM |
| [ADP]_m_ | Mitochondrial ADP concentration | 0.276 | mM |
| [NADH] | Mitochondrial NADH concentration | 5.403 | mM |
| Ψ_m_ | Inner mitochondrial membrane potential | -140.7 | mV |
| [ISOC] | Isocitrate concentration (mitochondrial) | 0.41 | mM |
| [αKG] | Α-ketoglutarate concentration (mitochondrial) | 8∙10^-4^ | mM |
| [SCoA] | Succinyl-CoA concentration (mitochondrial) | 0.362∙10^-3^ | mM |
| [­Suc] | Succinate concentration (mitochondrial) | 1.06∙10^-4^ | mM |
| [FUM] | Fumarate concentration (mitochondrial) | 0.0282 | mM |
| [MAL] | Malate concentration (mitochondrial) | 0.01316 | mM |
| [OAA] | Oxalacetate concentration (mitochondrial) | 2.367∙10^-4^ | mM |
| [FLV] | Flavoprotein concentration | 5.403 | mM |
| [AcCoA] | Acetyl CoA concentration | 1 | mM |

**b.** **Sarcoplasmic Reticulum (SR) and Ca^2+^ Handling**

The modeling of Ca^2+^ concentration in the cell is based on division of the cell into several inner compartments, each with different calcium buffering capacities. In this section, we model the buffering of all compartments and the intracellular calcium currents between compartments. The inner compartments modeled are the cytoplasm (annotated by “in”) and a SR uptake and release compartment (1, 3).

The model parameters are summarized in Table S3 and the initial values of the state variables are presented in Table S2.

*i. Uptake current of Ca^2+^ into the SR*

1. $I_{\mathrm{up}} = I_{up,max}\cdot\frac{{[ATP]}_{i}}{7.977}\frac{\frac{\left[ Ca^{2+} \right]_{i}}{K_{cy,Ca}}-\frac{K_{\mathrm{xcs}}^{2}\left[ Ca^{2+} \right]_{\mathrm{up}}}{K_{sr,Ca}}}{\frac{\left[ Ca^{2+} \right]_{i} + K_{cy,Ca}}{K_{cy,Ca}}+\frac{K_{\mathrm{xcs}}\left( \left[ Ca^{2+} \right]_{\mathrm{up}}+K_{sr,Ca} \right)}{K_{sr,Ca}}}$,

where $I_{up,max}$ is the maximal calcium uptake current into the SR, $K_{cy,Ca}$ is the equilibrium binding $Ca^{2+}$ concentration on the cytosolic side, $K_{sr,Ca}$ is the equilibrium binding $Ca^{2+}$ concentration in the uptake compartment of the SR side, $K_{\mathrm{xcs}}$ is a translocation constant and $\left[ Ca^{2+} \right]_{\mathrm{up}}$ is the Ca^2+^ in the uptake compartment of the SR.

*ii. Transfer current between the uptake and release compartments of the SR*

1. $I_{\mathrm{tr}} = \left( \left[ Ca^{2+} \right]_{\mathrm{up}} -\left[ Ca^{2+} \right]_{\mathrm{rel}} \right)\frac{2FV_{\mathrm{up}}}{\tau_{\mathrm{tr}}}$
2. $\frac{d\left[ Ca^{2+} \right]_{\mathrm{up}}}{\mathrm{dt}}=\frac{I_{\mathrm{up}}-I_{\mathrm{tr}}}{2V_{\mathrm{up}}F},$

where $\left[ Ca^{2+} \right]_{\mathrm{rel}}$ is the calcium concentration in the release compartment of the SR, $V_{\mathrm{up}}$ is the volume of the uptake compartment of the SR and $\tau_{\mathrm{tr}}$ is a transfer time constant.

*iii. Release current of Ca^2+^ from the SR*

1. $I_{\mathrm{rel}}=\alpha_{\mathrm{rel}}\left( \frac{F_{2}}{F_{2} + 0.25} \right)^{2}\left( \left[ \mathrm{Ca} \right]_{\mathrm{rel}} -\left[ \mathrm{Ca} \right]_{i} \right)$
2. $\frac{d\left[ Ca^{2+} \right]_{\mathrm{rel}}}{\mathrm{dt}}=\frac{I_{\mathrm{tr}}-I_{\mathrm{rel}}}{2V_{\mathrm{rel}}F} - 21\frac{dO_{\mathrm{Calse}}}{\mathrm{dt}}$
3. $\frac{dF_{1}}{\mathrm{dt}} = k_{\mathrm{recov}}F_{3}-k_{\mathrm{act}}F_{1}$
4. $\frac{dF_{2}}{\mathrm{dt}} = k_{\mathrm{act}}F_{1} - k_{\mathrm{inact}}F_{2}$
5. $\frac{dF_{3}}{\mathrm{dt}} = k_{\mathrm{inact}}F_{2} - k_{\mathrm{recov}}F_{3}$
6. $k_{\mathrm{act}}={240}^{*}e^{\frac{V - 20}{12.5}} + 203.8\left( \frac{\left[ Ca^{2+} \right]_{i}}{\left[ Ca^{2+} \right]_{i}+K_{M,rel}} \right)^{4}$
7. $k_{\mathrm{inact}}= 33.96 + 339.6\left( \frac{\left[ Ca^{2+} \right]_{i}}{\left[ Ca^{2+} \right]_{i}+K_{M,rel}} \right)^{4}$,

where $\alpha_{\mathrm{rel}}$ is the rate constant for calcium release, $V_{\mathrm{rel}}$ is the volume of the release compartment of the SR, $k_{\mathrm{recov}}$ is the rate constant for recovery of the calcium-dependent calcium release channels from their inactivated state and $K_{M,rel}$ is the equilibrium binding constant of SR $Ca^{2+}$ release gate for $\left[ Ca^{2+} \right]_{i}$. This constant takes different values in different compartments. Here we used the values in Aslanidi et al. (3).

1. $\frac{dO_{c}}{\mathrm{dt}}= 200000\left[ Ca^{2+} \right]_{i}\left( 1-O_{c} \right)-100\cdot O_{c}$
2. $\frac{dO_{\mathrm{TnCa}}}{\mathrm{dt}}=F_{\mathrm{kl}}\left[ Ca^{2+} \right]_{i}\left( 1-A-TT-U \right)+F_{\mathrm{kl}}\left[ Ca^{2+} \right]_{i}U-TTK_{-1}-A K_{-1}$
3. $\frac{dO_{\mathrm{Calse}}}{\mathrm{dt}}=(960\left[ Ca^{2+} \right]_{\mathrm{rel}}\left( 1-O_{\mathrm{Calse}} \right)-400\cdot O_{\mathrm{Calse}})/1000$
4. $\Phi_{\left[ Ca+2 \right]_{i}}= 0.08\frac{O_{\mathrm{TnCa}}}{\mathrm{dt}} + 0.045\frac{dO_{c}}{\mathrm{dt}}$
5. $\frac{d\left[ Ca^{2+} \right]_{i}}{\mathrm{dt}} =\frac{2I_{\mathrm{NaCa}}-I_{\mathrm{Ca},L}-I_{\mathrm{Ca},T}-I_{\mathrm{Cap}}-I_{\mathrm{Ca},b}-I_{\mathrm{up}}+I_{\mathrm{rel}}}{2V_{\mathrm{Ca}}F} - \Phi_{\left[ Ca^{2+} \right]_{i}}$,

where $\left[ Mg^{2+} \right]_{\mathrm{in}}$ is the intracellular magnesium concentration and $V_{\mathrm{Ca}}$is the intracellular volume for $Ca^{2+}$. The calcium buffers are $O_{c}$ – binding to calmodulin, $O_{\mathrm{TnCa}}$ – binding to troponin in the sarcomeres to activate the actin-myosin activity and $O_{\mathrm{Calse}}$ – binding to calsequestrin in the SR. Note that calmodulin and sarcomere activities directly affect the cytoplasmatic Ca^2+^ concentration via $\Phi_{\left[ Ca^{2+} \right]_{\mathrm{in}}}$, but as an SR buffer, calsequestrin affects only the SR Ca^2+^ concentrations. K_-1_ describes the dependence of Ca^2+^ affinity on the number of strong cross-bridges and $F_{\mathrm{kl}}$ is the rate constant of calcium binding to low-affinity troponin sites.

**Table S3: SR and Ca^2+^ handling parameters**

| **Units** | **Value** | **Description** | **Symbol** |
| --- | --- | --- | --- |
| pA | 6860 | Maximal Ca^2+^ uptake current into the SR | I_up,max_ |
| ms | 0.01 | Time transfer constant | $\tau_{tr}$ |
| [] | 0.045 | Equilibrium binding Ca^2+^ concentration on the cytosol side | K_cy,Ca_ |
| [] | 5 | Equilibrium binding Ca^2+^ concentration in the uptake compartment of the SR side | K_sr,Ca_ |
| [] | 0.2 | Translocation constant | K_xcs_ |
| pA/mM | 25000 | Rate constant for Ca^2+^ release | α_rel_ |
| 1/s | 0.815 | Rate constant for recovery of the Ca^2+^-dependent Ca^2+^ release channels from their inactivated state | k_recov_ |
| mM | 0.0003 | Equilibrium binding constant of SR Ca^2+^ release gate for [Ca^2+^]_in_ | K_M,rel_ |
| 1/mM/ms | 300000 | The rate constant of calcium binding to low-afﬁnity troponin sites | F_kl_ |

**c.** **Force generation and sarcomere energy consumption**

In the implementation we present here, we assume isometric contraction. The effect of sarcomere length on the force generated by the atrial cell is modeled based on (4).

The model parameters are summarized in Table S4 and the initial values of the state variables are presented in Table S2.

1. $\frac{dV_{e}}{\mathrm{dt}}=0$
2. $\frac{\mathrm{dSL}}{\mathrm{dt}}=-Ve$
3. $N_{\mathrm{XB}} = {10}^{-6}\left( SL - SL_{0} \right)N_{c}\left( TT+U \right)\frac{1000}{2}$
4. $K_{\mathrm{Ca}}=F_{k0} +\frac{F_{k1}{N_{\mathrm{XB}}}^{\mathrm{FN}}}{{F_{k05}}^{\mathrm{FN}} + {N_{\mathrm{XB}}}^{\mathrm{FN}}}$
5. $K_{-1} =\frac{F_{\mathrm{kl}}}{K_{\mathrm{Ca}}}$
6. $\frac{\mathrm{dA}}{\mathrm{dt}}=F_{\mathrm{kl}}\left[ Ca^{2+} \right]_{i}\left( 1-A-TT-U \right)-A(F_{f} + K_{-1}) + TT(F_{\mathrm{go}} + F_{\mathrm{gl}}V_{e})$
7. $\frac{\mathrm{dTT}}{\mathrm{dt}} = F_{f}A-TT\left( F_{\mathrm{go}}+F_{\mathrm{gl}}V_{e}+K_{-1} \right)+F_{\mathrm{kl}}\left[ Ca^{2+} \right]_{i}U$
8. $\frac{\mathrm{dU}}{\mathrm{dt}} = K_{-1}TT-\left( F_{\mathrm{go}}+F_{\mathrm{gl}}V_{e}+F_{\mathrm{kl}}\left[ Ca^{2+} \right]_{i} \right)U$
9. $N_{XB,ATP}=\frac{1.02}{1+\frac{K_{M_{\mathrm{ATP}}}}{\mathrm{AT}P_{i}} \left( 1+\frac{\mathrm{AD}P_{i}}{K_{M_{\mathrm{ADP}}}} \right)}$
10. $Force=F_{\mathrm{XB}}N_{XB,ATP}\frac{\left( SL - SL_{0} \right)}{2}\left( TT + U \right)N_{c}$
11. $\mathrm{Force}_{\mathrm{ATP}}=\frac{1.02}{1 + \left( \frac{K_{M_{\mathrm{ATP}}}}{\mathrm{AT}P_{i}} \right)*\left( 1 +\frac{2.6 - ATP_{i}}{K_{M_{\mathrm{ADP}}}} \right)}$_,_

where $SL_{0}$ is the minimal length of the sarcomere, $N_{c}$ is the reciprocal of the cross-section of the tissue, $F_{k0}$ is the cross-bridge-independent coefficient of Ca^2+^ affinity, $F_{k1}$ is the cooperativity coefficient, FN is the Hill coefficient, $F_{k,05}$ is the half-maximal cross-bridge Ca^2+^ affinity, $F_{f}$ is the cross-bridge turnover rate from the weak to the strong conformation, $F_{\mathrm{go}}$ is the cross-bridge weakening rate at isometric regime, $F_{\mathrm{gl}}$ is the mechanical feedback coefficient (describes the dependence of the XB weakening rate on the shortening velocity), $K_{M_{\mathrm{ATP}}}$ and $K_{M_{\mathrm{ADP}}}$ are constants representing the dependence of force generation on the energy state of the cell and $F_{\mathrm{XB}}$ is the unitary force per cross-bridge at the isometric regime.

1. $\mathrm{AT}P_{\mathrm{XB}} = ATP_{\mathrm{Max}}F_{f}A\cdot Force$
2. $V_{\mathrm{AM}}= ATP_{\mathrm{Max}}F_{f}A\cdot ForceATP$,

where $\mathrm{AT}P_{\mathrm{Max}}$ is the maximal ATP consumption by the sarcomeres.

**Table S4: Force generation and energy consumption parameters**

| **Units** | **Value** | **Description** | **Symbol** |
| --- | --- | --- | --- |
| µm | 0.8 | A constant coefﬁcient that describes the effect of the actin and myosin ﬁlament lengths on the single overlap length. | SL_0_ |
| 1/mm^2^ | 2∙10^13^ | The reciprocal of atrial myofiber cross-section area | N_C_ |
| 1/mM | 350 | The cross-bridge-independent coefﬁcient of calcium afﬁnity. | F_k0_ |
| 1/mM | 3000 | The cooperativity coefﬁcient. Describes the dependence of calcium afﬁnity on the number of strong cross-bridges | F_k1_ |
| [] | 3.5 | Hill coefficient | FN |
| 1/mm^3^ | 2.5·10^9^ | Half-maximal cross-bridge Ca^2+^ affinity | F_k,0.5_ |
| 1/ms | 40 | The cross-bridge turnover rate from the weak to the strong conformation | F_f_ |
| 1/ms | 30 | The cross-bridge weakening rate at isometric regime | F_g0_ |
| 1/m | 4.4·10^6^ | The mechanical feedback coefﬁcient. Describes the dependence of the XB weakening rate on the shortening velocity | F_g1_ |
| mN | 2·10^-9^ | The unitary force per cross-bridge at isometric regime | F_XB_ |
| mM | 0.0012 | Maximal ATP consumption by the sarcomeres | ATP_max_ |
| [] | 0.03 | Coefficient of force generation at the energy state of the cell | $K_{M_{\mathrm{ATP}}}$ |
| [] | 0.26 | Coefficient of force generation at the energy state of the cell | $K_{M_{\mathrm{ADP}}}$ |

**d.** **Mitochondrial energy metabolism**

This part of the model describes atrial mitochondrial metabolism, mitochondrial Ca^2+^ dynamics and oxygen consumption. It includes the tricarboxylic acid (TCA) cycle and its regulating enzymes, oxidative phosphorylation and the interplay between them. The calculated energy demand is the consumption of the main dynamic processes that are mainly influenced by action potentials. Other energy-demanding processes which are governed by other stimuli, such as neuronal or hormonal activations, are not modeled.

The model parameters of the TCA cycle are summarized in Table S5, oxidative phosphorylation in Table S6, and the initial values of the state variables in Table S2. As the models used in this section are based on rat ventricular cell data, model parameters were adjusted to fit data from rabbit atria based on measurements with 1 Hz stimulation frequency and then the model was tested against data from cells with 3 Hz stimulation frequency.

*i. Mitochondrial energetics and excitation contraction coupling*

Excitation contraction coupling and mitochondrial energetics are linked through ATP, adenosine diphosphate (ADP), creatine, creatine phosphate and mitochondrial and cytoplasmic Ca^2+^ concentrations. ATP_i_ (EC coupling linked) is the internal ATP pool within the cytosplasm. The total concentration of adenine nucleotides is constant (C_A_) is the same. Therefore, the following equation applies:

1. $[{ADP]}_{i}=C_{A}-\left[ \mathrm{ATP} \right]_{i}$.

*ii. The TCA cycle*

The TCA cycle was modeled as a series of reactions incorporating regulatory feedback loops similar to (5). It consists of eight steps catalyzed by eight different enzymes. This cycle harnesses the available chemical energy of acetyl CoA into the reducing power of NADH. The following equations model the main control of the TCA cycle by cellular energy demands, as represented by mitochondrial Ca^2+^ concentration changes and the link between glycolysis and the TCA cycle. The link to glycolysis is represented by the Ca^2+^-controlled pyruvate dehydrogenase activity, which serves as the source of acetyl CoA for the cycle.

We modified this energetic equation in (5) to include regulation by mitochondrial Ca^2+^ ([Ca^2+^]_m_) similar to (6).

1. $V_{\mathrm{PDH}}=k_{\mathrm{PDH}}\cdot C_{\mathrm{PYR}}\cdot(\frac{\left[ Ca^{2+} \right]_{m}}{\left[ Ca^{2+} \right]_{m}+k_{\mathrm{CaAcCoA}}})$,

where $k_{\mathrm{PDH}}$ is the is the catalytic constant of pyruvate dehydrogenase (PDH), $C_{\mathrm{PYR}}$ is the pyruvate concentration and $k_{\mathrm{CaAcCoA}}$ is the Michaelis constant representing the Ca^2+^ regulatory effect on PDH.

1. $V_{\mathrm{CS}}=k_{\mathrm{cat}}^{\mathrm{Cs}}E_{T}^{\mathrm{CS}}{(1+\frac{K_{M}^{\mathrm{AcCoA}}}{\left[ \mathrm{AcCoA} \right]}+\frac{K_{M}^{\mathrm{OAA}}}{\left[ \mathrm{OAA} \right]}+\frac{K_{M}^{\mathrm{AcCoA}}}{[AcCoA]}\cdot\frac{K_{M}^{\mathrm{OAA}}}{\left[ \mathrm{OAA} \right]})}^{-1}$,

where [AcCoA] is acetyl CoA concentration, OAA is oxaloacetate concentration, $k_{\mathrm{cat}}^{\mathrm{Cs}}$ is the catalytic constant of citrate synthase (CS), $E_{T}^{\mathrm{CS}}$ is CS concentration, $K_{M}^{\mathrm{AcCoA}}$ is the Michaelis constant of AcCoA, and K_M_^OAA^ is the Michaelis constant of OAA.

1. $\frac{d[AcCoA]}{\mathrm{dt}}=V_{\mathrm{PDH}}-V_{\mathrm{CS}}$.
2. $V_{\mathrm{ACO}}=k_{f}^{\mathrm{ACO}}(\left[ \mathrm{CIT} \right]-\frac{\left[ \mathrm{ISOC} \right]}{K_{E}^{\mathrm{ACO}}})$,

where $k_{f}^{\mathrm{ACO}}$ is the forward rate constant of aconitase (ACO), $K_{E}^{\mathrm{ACO}}$ is the equilibrium constant of ACO, [CIT] is the concentration of citrate and [ISOC] is the concentration of isocitrate.

1. $f_{a}^{\mathrm{IDH}}{=\left[ \left( 1+\frac{[{ADP]}_{m}}{K_{\mathrm{ADP}}^{a}} \right)\left( 1+\frac{[{\mathrm{Ca}^{2+}]}_{m}}{K_{\mathrm{Ca}}^{a}} \right) \right]}^{-1}$
2. $f_{i}^{\mathrm{IDH}}=\left( 1+\frac{\left[ \mathrm{NADH} \right]}{K_{i,NADH}} \right)$
3. $V_{\mathrm{IDH}}=k_{\mathrm{cat}}^{\mathrm{IDH}}E_{T}^{\mathrm{IDH}}\left[ 1+\frac{\left[ H^{+} \right]}{k_{h,1}}+\frac{k_{h,2}}{\left[ H^{+} \right]}+f_{i}^{\mathrm{IDH}}\left( \frac{K_{M}^{\mathrm{NAD}}}{\left[ \mathrm{NAD} \right]} \right)+f_{a}^{\mathrm{IDH}}\left( \frac{K_{M}^{\mathrm{ISOC}}}{\left[ \mathrm{ISOC} \right]} \right)^{\mathrm{Ni}}+f_{a}^{\mathrm{IDH}}f_{i}^{\mathrm{IDH}}\left( \frac{K_{M}^{\mathrm{NAD}}}{\left[ \mathrm{NAD} \right]} \right)\left( \frac{K_{M}^{\mathrm{ISOC}}}{\left[ \mathrm{ISOC} \right]} \right)^{\mathrm{Ni}} \right]^{-1}$,

where $K_{\mathrm{ADP}}^{a}$ is the activation constant by ADP, $K_{\mathrm{Ca}}^{a}$ is the activation constant for Ca^2+^, $K_{i,NADH}$ is the inhibition constant by NADH, $k_{\mathrm{cat}}^{\mathrm{IDH}}$ is the isocitrate dehydrogenase (IDH) rate constant, $E_{T}^{\mathrm{IDH}}$is IDH concentration, $K_{M}^{\mathrm{ISOC}}$ is the Michaelis constant for isocitrate, $K_{M}^{\mathrm{NAD}}$ is the Michaelis constant for NAD^+^, $\mathrm{Ni}$ is the isocitrate cooperativity, $\left[ H^{+} \right]$ is matrix proton concentration, and $k_{h,1}$ and $k_{h,2}$ are the ionization constants of IDH.

1. $\frac{d[ISOC]}{\mathrm{dt}}=V_{\mathrm{ACO}}-V_{\mathrm{IDH}}$.
2. $f_{a}^{\mathrm{KGDH}}=\left[ \left( 1+\frac{{{[Mg}^{2+}]}_{m}}{K_{D}^{\mathrm{Mg}^{2+}}} \right)\left( 1+\frac{{{[Ca}^{2+}]}_{m}}{K_{D}^{\mathrm{Ca}^{2+}}} \right) \right]^{-1}$
3. $V_{\mathrm{KGDH}}=K_{KGDH}\frac{k_{\mathrm{cat}}^{\mathrm{KGDH}}E_{T}^{\mathrm{KGDH}}}{\frac{1}{5}+f_{a}^{\mathrm{KGDH}}\left( 100\cdot\frac{k_{M}^{\alpha KG}}{\left[ \alpha KG \right]} \right)^{n_{a}\mathrm{KG}}+f_{a}^{\mathrm{KGDH}}\left( \frac{k_{M}^{NAD\_new}}{\left[ \mathrm{NAD} \right]\cdot0.3} \right)}$,

where $K_{D}^{\mathrm{Mg}^{2+}}$ is the activation constant for Mg^2+^, $K_{D}^{\mathrm{Ca}^{2+}}$ is the activation constant for Ca^2+^, [$\alpha KG$] is the alpha-ketoglutarate concentration, $E_{T}^{\mathrm{KGDH}}$ is the alpha-ketoglutarate dehydrogenase (KGDH) concentration, $k_{\mathrm{cat}}^{\mathrm{KGDH}}$is the KGDH rate constant, $k_{M}^{NAD\_new}$ is the Michaelis constant for NAD^+^, $k_{M}^{\alpha KG}$ is the Michaelis constant for αKG, $n_{a}\mathrm{KG}$ is the Hill coefficient of KGDH for αKG and ${[Mg}^{2+}]$ is the Mg^2+^ concentration in the mitochondria.

1. $\frac{d[\alpha KG]}{\mathrm{dt}}=V_{\mathrm{IDH}}-V_{\mathrm{KGDH}}$.
2. $V_{\mathrm{SCL}}=k_{f}^{\mathrm{SCL}}(\left[ \mathrm{SCoA} \right]\left[ \mathrm{ADP} \right]_{m}-\frac{\left[ \mathrm{Suc} \right][{ATP]}_{m}C_{\mathrm{CoA}}}{K_{E}^{\mathrm{SCL}}})$,

where $k_{f}^{\mathrm{SCL}}$ is the forward rate constant of SCL, $[SCoA]$ is the succinyl CoA concentration, [Suc] is the succinyl concentration and $K_{E}^{\mathrm{SCL}}$ is the SCoA reaction equilibrium constant.

1. $\frac{d[SCoA]}{\mathrm{dt}}=V_{\mathrm{KGDH}}-V_{\mathrm{SCL}}$
2. $V_{\mathrm{SDH}}=K_{SDH}\frac{k_{\mathrm{cat}}^{\mathrm{SDH}}E_{T}^{\mathrm{SDH}}}{1+\frac{K_{M}^{\mathrm{Suc}}}{\left[ \mathrm{Suc} \right]}\left( 1+\frac{\left[ \mathrm{OAA} \right]}{K_{I,sdh}^{\mathrm{OAA}}} \right)\left( 1+\frac{\left[ \mathrm{FUM} \right]}{K_{i}^{\mathrm{FUM}}} \right)}$,

where $k_{\mathrm{cat}}^{\mathrm{SDH}}$ is the SDH rate constant, $E_{T}^{\mathrm{SDH}}$ is the SDH enzyme concentration, $K_{M}^{\mathrm{Suc}}$ is the succinate Michaelis constant, $K_{i}^{\mathrm{FUM}}$ is the fumarate (FUM) inhibition constant, and $I$ is the oxaloacetate (OAA) inhibition constant.

1. $\frac{d[Suc]}{\mathrm{dt}}=V_{\mathrm{SL}}-V_{\mathrm{SDH}}$
2. $V_{\mathrm{FH}}=k_{f}^{\mathrm{FH}}(\left[ \mathrm{FUM} \right]-\frac{\left[ \mathrm{MAL} \right]}{K_{E}^{\mathrm{FH}}})$,

where $k_{f}^{\mathrm{FH}}$ is the FH forward rate constant, $K_{E}^{\mathrm{FH}}$ if the FH equilibrium constant, [FUM] is fumarate concentration and [MAL] is malate concentration.

1. $\frac{d\left[ \mathrm{FUM} \right]}{\mathrm{dt}}=V_{\mathrm{SDH}}-V_{\mathrm{FH}}$
2. $f_{h.a}={(1+\frac{{[H}^{+}]}{k_{h1}}+\frac{{{[H}^{+}]}^{2}}{k_{h1}k_{h2}})}^{-1}+k_{\mathrm{offest}}$
3. $f_{h,i}=\left( 1+\frac{k_{h3}}{{[H}^{+}]}+\frac{k_{h3}k_{h4}}{{{[H}^{+}]}^{2}} \right)^{-2}$
4. $V_{\mathrm{MDH}}=K_{MDH}\frac{k_{\mathrm{cat}}^{\mathrm{MDH}}E_{T}^{\mathrm{MDH}}f_{h,a}f_{h,i}}{1+\frac{K_{M}^{\mathrm{MAL}}}{\left[ \mathrm{MAL} \right]}\left( 1+\frac{\left[ \mathrm{OAA} \right]}{K_{i}^{\mathrm{OAA}}} \right)+\frac{K_{M}^{NAD\_x}}{[NAD]}+\frac{K_{M}^{\mathrm{MAL}}}{\left[ \mathrm{MAL} \right]}(1+\frac{\left[ \mathrm{OAA} \right]}{K_{i}^{\mathrm{OAA}}})\frac{K_{M}^{NAD\_x}}{[NAD]}}$ ,

where $k_{h1}$, $k_{h2}$, $k_{h3}$ and $k_{h4}$ are MAL dehydrogenase (MDH) ionization constants, $k_{\mathrm{offest}}$ is a pH independent term in the pH activation factor of MDH, $k_{\mathrm{cat}}^{\mathrm{MDH}}$ is the MDH rate constant, $E_{T}^{\mathrm{MDH}}$ is the total MDH enzyme concentration, $K_{M}^{\mathrm{MAL}}$ is the malate Michaelis constant, $K_{i}^{\mathrm{OAA}}$ is the OAA inhibition constant, $K_{M}^{NAD\_x}$ is the NAD^+^ Michaelis constant, [H^+^] is the proton matrix concentration, and [NAD] is the  nicotinamide adenine dinucleotide concentration

1. $\frac{d[MAL]}{\mathrm{dt}}=V_{\mathrm{FH}}-V_{\mathrm{MDH}}$
2. $\frac{d\left[ \mathrm{OAA} \right]}{\mathrm{dt}}=V_{\mathrm{MDH}}-V_{\mathrm{CS}}$

In summary, as we assumed that the TCA cycle is closed from the point of view of carbon intermediates, a conservation equation relating all TCA metabolites is shown here. Thus, the level of CIT is the result of the balance of all other intermediates in the cycle as follows:

1. $\left[ \mathrm{CIT} \right]=C_{\mathrm{Kint}}-\left( \left[ \mathrm{ISOC} \right]+\left[ \alpha KG \right]+\left[ \mathrm{SCoA} \right]+\left[ \mathrm{Suc} \right]+\left[ \mathrm{FUM} \right]+\left[ \mathrm{MAL} \right]+\left[ \mathrm{OAA} \right] \right)$

$C_{\mathrm{Kint}}$ is the sum of the concentration of the TCA cycle intermediate.

**Table S5: TCA cycle parameters**

| 1/ms | 0.4857 | Catalytic constant of CS | k_cat_^CS^ |
| --- | --- | --- | --- |
| mM | 0.4 | CS concentration | E_T_ ^CS^ |
| mM | 1.26 | AcCoA Michaelis constant | K_M_^AcCoA^ |
| mM | 6.4·10^-4^ | OAA Michaelis constant | K_M_^OAA^ |
| mM | 1 | Sum of TCA cycle intermediate concentration | C_Kint_ |
| 1/ms | 0.2587 | ACO forward rate constant | k_f_^ACO^ |
| [] | 0.9 | ACO equilibrium constant | K_E_^ACO^ |
| [] | 486.99 | ACO coefficient | K_ACO_ |
| mM | 0.62 | ADP activation constant | K^a^ _ADP_ |
| mM | 0.0005 | Ca^2+^ activation constant | K^a^ _Ca_ |
| mM | 0.19 | NADH inhibition constant | K_i,NADH_ |
| 1/ms | 15.3673 | IDH rate constant | k^IDH^ _cat_ |
| mM | 0.109 | IDH concentration | E_T_ ^IDH^ |
| [] | 512.2446 | IDH coefficient | K_IDH_ |
| mM | 2.5·10^-5^ | Matrix proton concentration | [H^+^] |
| mM | 8.1·10^-5^ | Ionization constant of IDH | k_h,1_ |
| mM | 5.98·10^-5^ | Ionization constant of IDH | k_h,2_ |
| mM | 1.52 | Isocitrate Michaelis constant | K_M_ ^ISOC^ |
| [] | 2 | Isocitrate cooperativity | N_i_ |
| mM | 0.923 | NAD^+^ Michaelis constant | K_M_ ^NAD^ |
| mM | 0.0308 | Mg^2+^ Activation constant | K_D_ ^Mg2+^ |
| mM | 6.35·10^-5^ | Ca^2+^ Activation constant | K_D_ ^Ca2+^ |
| mM | 0.5 | KGDH concentration | E_T_ ^KGDH^ |
| 1/ms | 0.254828 | KGDH rate constant | k_cat_ ^KGDH^ |
| mM | 1.94 | αKG Michaelis constant | K_M_ ^αKG^ |
| mM | 38.7 | NAD Michaelis constant | K_M_ ^NAD_new^ |
| [] | 0.48 | Hill coefficient of KGDH for αKG | n_αKG_ |
| mM | 0.4 | Mg^2+^ mitochondrial concentration | [Mg^2+^]_m_ |
| 1/mM·ms | 238.7 | SL forward rate constant | k_f_^SCL^ |
| [] | 3.115 | SL reaction equilibrium constant | K_E_^SCL^ |
| mM | 2·10^-4^ | Coenzyme A concentration | C_CoA_ |
| 1/ms | 13.854 | SDH rate constant | k_cat_ ^SDH^ |
| mM | 0.5 | SDH enzyme concentration | E_T_ ^SDH^ |
| mM | 0.03 | Succinate Michaelis constant | K_M_^Suc^ |
| mM | 1.3 | Fumarate inhibition constant | K_i_^FUM^ |
| mM | 0.15 | Oxaloacetate inhibition constant | K_i,sdh_^OAA^ |
| 1/ms | 1.5856 | FH forward rate constant | k_f_ ^FH^ |
| [] | 1 | FH equilibrium constant | K_E_^FH^ |
| mM | 1.13·10^-5^ | MDH ionization constant | k_h1_ |
| mM | 26.7 | MDH ionization constant | k_h2_ |
| mM | 6.68·10^-9^ | MDH ionization constant | k_h3_ |
| mM | 5.62·10^-6^ | MDH ionization constant | k_h4_ |
| [] | 55.209 | pH-independent term in the pH activation factor of MDH | k_offset_ |
| 1/ms | 55.7115 | MDH rate constant | k_cat_ ^MDH^ |
| mM | 0.154 | Total MDH enzyme concentration | E_T_ ^MDH^ |
| mM | 1.493 | Malate Michaelis constant | K_M_^MAL^ |
| mM | 3.1·10^-3^ | Oxaloacetate inhibition constant | K_i_^OAA^ |
| mM | 0.2244 | NAD^+^ Michaelis constant | K_M_^NAD_x^ |
| mM | 0.2∙10^-4^ | Ca^2+^-Acetyl CoA Michaelis constant | k_Ca,AcCoA_ |
| mM/s | 0.3298125 | Pyruvate dehydrogenase rate constant | k_PDH_ |
| mM | 0.12 | Pyruvate concentration | C_PYR_ |
| mM | 1.5 | Total sum of mitochondrial adenine nucleotides | C_A_ |

*iii The respiration-driven proton pump*

The model is based on (5) with modification as indicated below:

1. $\Delta\mu_{H}=-2.303\frac{\mathrm{RT}}{F}\Delta pH+\Delta\Psi_{m}$
2. $V_{\mathrm{He}}=6\rho^{\mathrm{res}}\frac{\left( r_{a}\exp\left( \frac{A_{\mathrm{res}}F}{\mathrm{RT}} \right)-\left( r_{a}+r_{b} \right)\exp\left( \frac{g6F\Delta\mu_{H}}{\mathrm{RT}} \right) \right)}{(\left( 1+r_{1}\exp\left( \frac{A_{\mathrm{res}}F}{\mathrm{RT}} \right) \right)\exp\left( \frac{6F\Delta\Psi_{B}}{\mathrm{RT}} \right)+(r_{2}+r_{3}\exp\left( \frac{A_{\mathrm{res}}F}{\mathrm{RT}} \right))\exp\left( \frac{g6F\Delta\mu_{H}}{\mathrm{RT}} \right))}$ ,

where $r_{a}$, $r_{b}$,$r_{1}$, $r_{2}$ and $r_{3}$ are the sum of products of rate constants, $\rho^{\mathrm{res}}$ is the concentration of electron carriers of respiratory complexes I-III-IV, $\Delta\Psi_{B}$ is the phase boundary potential, $g$ is the voltage correction factor, and ΔpH is the pH gradient across the inner membrane.

Mitochondrial NAD^+^ is assumed to be conserved according to the following relation:

1. $\left[ \mathrm{NAD} \right]=C_{\mathrm{PN}}-\left[ \mathrm{NADH} \right]$ ,

where $C_{\mathrm{PN}}$ is the total sum of mitochondrial pyridine nucleotides.

1. $A_{res\_NADH}=\frac{\mathrm{RT}}{F}ln(K_{\mathrm{res}}\sqrt{\frac{\left[ \mathrm{NADH} \right]}{[NAD]}})$
2. $A_{res\_FLV}=\frac{\mathrm{RT}}{F}ln(K_{\mathrm{res}}\sqrt{\frac{\left[ \mathrm{FADH}_{2} \right]}{\left[ FAD \right]}})$
3. $\left[ \mathrm{FAD} \right]=\mathrm{Tot}_{\mathrm{FAD}}-\left[ \mathrm{FADH}_{2} \right] ,$

where $K_{\mathrm{res}}$ is the equilibrium constant of respiration, $\left[ \mathrm{FADH}_{2} \right]$ is reduced FAD concentration, $\left[ \mathrm{FADH} \right]$ is oxidized FAD concentration and Tot_FLV_ is the total FADH_2_ and FAD concentrations.

As the reducing reagents, FADH_2_ and NADH are created in the TCA cycle in 1:4 ratio (FADH_2_:NADH) and we assume that their consumption by the electron transfer chain follows the same ratio. This means that 1/5 of oxygen consumption is attributed to FADH_2_-fueled and 4/5 to NADH-fueled electron transfer cycles:

1. $A_{\mathrm{res}}=\frac{4}{5}A_{res\_NADH}+\frac{1}{5}A_{res\_FLV}$.

In a modification of the original formulation in (5), it is also considered that the complex II electrons, input by SUC through FADH_2_ to the respiratory chain:

1. $A_{\mathrm{res}\left( F \right)}=\frac{\mathrm{RT}}{F}ln(K_{\mathrm{res}\left( F \right)}\sqrt{\frac{\left[ \mathrm{FADH}_{2} \right]}{[FAD]})}$
2. $V_{\mathrm{He}\left( F \right)}=4\rho^{\mathrm{res}\left( F \right)}\frac{\left( r_{a}\exp\left( \frac{A_{\mathrm{res}\left( F \right)}F}{\mathrm{RT}} \right)-\left( r_{a}+r_{b} \right)\exp\left( \frac{g6F\Delta\mu_{H}}{\mathrm{RT}} \right) \right)}{(\left( 1+r_{1}\exp\left( \frac{A_{\mathrm{res}\left( F \right)}F}{\mathrm{RT}} \right) \right)\exp\left( \frac{6F\Delta\Psi_{B}}{\mathrm{RT}} \right)+(r_{2}+r_{3}\exp\left( \frac{A_{\mathrm{res}\left( F \right)}F}{\mathrm{RT}} \right))\exp\left( \frac{g6F\Delta\mu_{H}}{\mathrm{RT}} \right))}$ .

The flux of protons driven by FADH_2_ oxidation (V_He(F)_) has the same form as V_He_, except for the adjustment of the redox potential and the H^+^ stoichiometry. $\rho^{\mathrm{res}\left( F \right)}$ is the concentration of electron carriers (respiratory complexes II-III-IV) and $K_{\mathrm{res}\left( F \right)}$ is the equilibrium constant of FADH_2_ oxidation.

The regulation of oxidative phosphorylation by the cellular energy demands is modeled using a push and pull mechanism. ADP activates enzymes in the Krebs cycle and thereby “pushes” the respiratory flux toward ATP generation (6). In parallel, ADP controls ATP synthase by its availability, and thereby “pulls” ATP production by increasing respiratory flux (7):

1. $pull=\frac{V_{ATPase}}{k_{ATPase}}$
2. $V_{O2}=\rho^{\mathrm{res}}pull\frac{(\left( r_{a}+r_{c1}\exp\left( \frac{6F\Delta\Psi_{B}}{\mathrm{RT}} \right) \right)\exp\left( \frac{A_{\mathrm{res}}F}{\mathrm{RT}} \right)-r_{a}\exp\left( \frac{g6F\Delta\mu_{H}}{\mathrm{RT}} \right)+r_{c2}\exp\left( \frac{A_{\mathrm{res}}F}{\mathrm{RT}} \right)\exp\left( \frac{g6F\Delta\mu_{H}}{\mathrm{RT}} \right))}{(\left( 1+r_{1}\exp\left( \frac{A_{\mathrm{res}}F}{\mathrm{RT}} \right) \right)\exp\left( \frac{6F\Delta\Psi_{B}}{\mathrm{RT}} \right)+\left( r_{2}+r_{3}\exp\left( \frac{A_{\mathrm{res}}F}{\mathrm{RT}} \right) \right)\exp\left( \frac{g6F\Delta\mu_{H}}{\mathrm{RT}} \right))}$ ,

where $r_{c1}$ and$r_{c2}$ are the sum of products of rate constants and k_ATPase_ is defined as the coupling coefficient representing the pull effect of the ATP synthase on the activity of the electron transfer chain.

1. $\frac{d[NADH]}{\mathrm{dt}}=-\frac{4}{5}V_{o_{2}}+V_{\mathrm{IDH}}+V_{\mathrm{KGDH}}+V_{\mathrm{MDH}}+V_{\mathrm{PDH}}$
2. $\frac{d[FLV]}{\mathrm{dt}}=V_{\mathrm{SDH}}-\frac{1}{5}V_{o_{2}}.$

*iv. F_1_F_0_-ATPase*

According to the concept of respiratory control, mitochondrial function is governed by the availability of ADP and P_i_. The chemiosmotic hypothesis dictates that ΔΨ_m_ is lowered by an H^+^ influx, which drives the production of ATP by F_1_F_o_-ATPase.

1. $V_{\mathrm{ATPase}}=-{30\cdot\rho}^{F1}\frac{(({10}^{2}p_{a}+p_{c1}\exp\left( \frac{3F\Delta\Psi_{B}}{\mathrm{RT}} \right))\exp\left( \frac{\mathrm{FA}_{F1}}{\mathrm{RT}} \right)-(p_{a}\exp\left( \frac{3F\Delta\mu_{H}}{\mathrm{RT}} \right)+p_{c2}\exp\left( \frac{\mathrm{FA}_{F1}}{\mathrm{RT}} \right)\exp\left( \frac{3F\Delta{\Psi\mu}_{H}}{\mathrm{RT}} \right)))}{\left( \left( 1+p_{1}\exp\left( \frac{\mathrm{FA}_{F1}}{\mathrm{RT}} \right)\exp\left( \frac{3F\Delta\Psi_{B}}{\mathrm{RT}} \right) \right)+\left( p_{2}+p_{3}\exp\left( \frac{\mathrm{FA}_{F1}}{\mathrm{RT}} \right) \right)\exp\left( \frac{3F\Delta\mu_{H}}{\mathrm{RT}} \right) \right)}\cdot(1-\exp\left( -\frac{\mathrm{Ca}_{m}}{\mathrm{KCaATP}} \right))$
2. $V_{\mathrm{Hu}}=-3\rho^{F1}\frac{({10}^{2}p_{a}\left( 1+\exp\left( \frac{\mathrm{FA}_{F1}}{\mathrm{RT}} \right) \right)-(p_{a}+p_{b})\exp\left( \frac{3F\Delta\mu_{H}}{\mathrm{RT}} \right)}{(\left( 1+p_{1}\exp\left( \frac{\mathrm{FA}_{F1}}{\mathrm{RT}} \right) \right)\exp\left( \frac{3F\Delta\Psi_{B}}{\mathrm{RT}} \right)+(p_{2}+p_{3}\exp\left( \frac{\mathrm{FA}_{F1}}{\mathrm{RT}} \right))\exp\left( \frac{3F\Delta\mu_{H}}{\mathrm{RT}} \right))}$
3. $A_{F1}=\frac{\mathrm{RT}}{F}\ln\left( K_{F1}\frac{[{ATP]}_{m}}{[{ADP]}_{m}\mathrm{Pi}} \right)$
4. ${[ATP]}_{m}=C_{A}-[{ADP]}_{m}$ ,

where $p_{a}$, $p_{b}$, $p_{c1}$,$p_{c2}$, $p_{1}$, $p_{2}$ and $p_{3}$ are the sum of products of rate constants, $\rho^{F1}$ is the F_1_F_0_-ATPase concentration, $K_{F1}$ is the equilibrium constant of ATP hydrolysis, $\mathrm{Pi}$ is the inorganic phosphate concentration and $C_{A}$ is the total sum of mitochondrial adenine nucleotides.

*v. Adenine nucleotide translocator (ANT) and proton leak*

To complete the description of the major membrane oxidative phosphorylation-associated processes, the exchange of adenine nucleotides across the mitochondrial membrane as well as the proton leak are considered, based on (5). The ANT equation is modeled according to a sequential mechanism of the carrier. V_ANT_, the flux of ANT-mediated exchange between cytosolic ADP and matrix ATP, is considered to be electrogenic and dependent on the gradients of both ATP and ADP across the inner mitochondrial membrane as follows:

1. $V_{\mathrm{ANT}}=V_{max,ANT}\frac{0.75\left( 1-\frac{0.25\left[ \mathrm{ATP} \right]_{i}\times0.45[{ADP]}_{m}}{0.17\left[ \mathrm{ADP} \right]_{i}\times0.025[{ATP]}_{m}} \right)\exp\left( -\frac{F\Delta\Psi_{m}}{\mathrm{RT}} \right)}{(1+0.0001\cdot\frac{0.25\left[ \mathrm{ATP} \right]_{i}}{0.225\left[ \mathrm{ADP} \right]_{i}}exp(\frac{{-h}^{\mathrm{ANT}}F\Delta\Psi_{m}}{\mathrm{RT}})(\frac{0.45\left[ \mathrm{ADP} \right]_{m}}{0.025\left[ \mathrm{ATP} \right]_{m}})}$ ,

where $V_{max,ANT}$ is the maximal ANT rate and $h^{\mathrm{ANT}}$ if the fraction of $\Delta\Psi_{m}$.

The proton leak is considered to be a linear function of the Δµ_H_ through a proportionally constant given by the H^+^ conductance g_h_:

1. $V_{\mathrm{HLeak}}=g_{h}\Delta\mu_{H}$ ,

where $g_{h}$ is the ionic conductance of the inner membrane.

1. $\frac{{d[ADP]}_{m}}{\mathrm{dt}}=V_{\mathrm{ANT}}-V_{\mathrm{ATPase}}-V_{\mathrm{SCL}}$
2. $\frac{{d\left[ \mathrm{ATP} \right]}_{i}}{\mathrm{dt}}=V_{\mathrm{ANT}}\frac{V_{\mathrm{mito}}}{V_{\mathrm{myo}}}-0.5I_{\mathrm{up}}-\left( I_{\mathrm{CaP}}+I_{\mathrm{NaK}} \right)\frac{A_{\mathrm{cap}}}{V_{\mathrm{myo}}F}-V_{\mathrm{AM}}$

**Table S6: Oxidative phosphorylation parameters**

| 1/ms | 6.394·10^-13^ | Sum of products of rate constants | r_a_ |
| --- | --- | --- | --- |
| 1/ms | 1.76·10^-16^ | Sum of products of rate constants | r_b_ |
| 1/ms | 2.656·10^-17^ | Sum of products of rate constants | r_c1_ |
| 1/ms | 8.632·10^-30^ | Sum of products of rate constants | r_c2_ |
| [] | 3.65475∙10^-14^ | Sum of products of rate constants | r_1_ |
| [] | 1.728·10^-9^ | Sum of products of rate constants | r_2_ |
| [] | 1.059·10^-26^ | Sum of products of rate constants | r_3_ |
| mM | 15.7894 | Electron carrier concentration (respiratory complexes I-III-IV) | ρ^res^ |
| [] | 1.35·10^18^ | Equilibrium constant of respiration | K_res_ |
| mM | 3.75·10^-4^ | Electron carrier concentration (respiratory complexes II-III-IV) | ρ^resF^ |
| mV | 1500 | Phase boundary potential | Ψ_B_ |
| [] | 0.85 | Voltage correction factor | G |
| [] | 5.765·10^13^ | Equilibrium constant of FADH_2_ oxidation | K_resF_ |
| mM | 1.26 | Total FADH and FADH2 concentration | Tot_FLV_ |
| 1/ms | 1.656·10^-8^ | Sum of products of rate constants | p_a_ |
| 1/ms | 3.337·10^-10^ | Sum of products of rate constants | p_b_ |
| 1/ms | 9.651·10^-7^ | Sum of products of rate constants | p_c1_ |
| 1/ms | 7.739·10^-7^ | Sum of products of rate constants | p_c2_ |
| [] | 1.346·10^-8^ | Sum of products of rate constants | p_1_ |
| [] | 7.739·10^-7^ | Sum of products of rate constants | p_2_ |
| [] | 6.65·10^-7^ | Sum of products of rate constants | p_3_ |
| mM | 1.5·10^-3^ | F1-F0 ATPase concentration | ρ_F1_ |
| [] | 1.71·10^6^ | ATP hydrolysis equilibrium constant | K_F1_ |
| mM | 2 | Inorganic phosphate concentration | Pi |
| mM/ms | 1.44918411·10^-5^ | ANT maximal rate | V_ANT,max_ |
| [] | 0.05 | Fraction of ΔΨ_m_ | h^ANT^ |
| mM/(ms·mV) | 10^-8^ | Inner membrane ionic conductance | g_H_ |
| [] | -0.6 | pH gradient across the inner membrane | ΔpH |
| mM | 10 | Total sum of mitochondrial pyridine nucleotides | C_PN_ |

**e.** **Mitochondrial Ca^2+^ dynamics**

This model assumes that mitochondrial Ca^2+^ dynamics is controlled by three processes (8): Ca^2+^  influx through the Ca^2+^ uniporter, Ca^2+^ efflux through the NCX and Ca^2+^ buffering.

The model parameters of mitochondrial Ca^2+^ dynamics are summarized in Table S7 and the initial values of the state variables are sumarized in Table S2.

*i. Ca^2+^ uniporter*

The mitochondrial Ca^2+^ uniporter depends on the electrochemical driving force of Ca^2+^. Thus, Ψ_m_ and extracellular Ca^2+^ concentration are the primary determinants of the uniporter flux. The uniporter is assumed to be an ion channel permeable only to Ca^2+^.

The uniporter flux (J_uni_) can be described by:

1. $J_{\mathrm{uni}}=P_{\mathrm{Ca}}\frac{Z_{\mathrm{Ca}}\Psi_{m}F}{\mathrm{RT}}\frac{\alpha_{m}\left[ \mathrm{Ca}^{2+} \right]_{m}\exp\left( \frac{-Z_{\mathrm{Ca}}\Psi_{m}F}{\mathrm{RT}} \right)-\alpha_{e}\left[ \mathrm{Ca}^{2+} \right]_{i}}{\exp\left( \frac{-Z_{\mathrm{Ca}}\Psi_{m}F}{\mathrm{RT}} \right)-1}$ ,

where $z_{\mathrm{Ca}}$ is Ca^2+^ valence, $P_{\mathrm{Ca}}$ is the Ca^2+^ uniporter permeability, $\alpha_{m}$is mitochondrial Ca^2+^ activity coefficient and $\alpha_{e}$ is the extra-mitochondrial Ca^2+^ activity coefficient.

*ii. Na^+^/Ca^2+^ exchanger*

The NCX flux (J_NC_) can be described by:

1. $J_{\mathrm{NC}}=\frac{V_{\mathrm{NC}}\left( \exp\left( \frac{0.5{\Delta\Psi}_{m}F}{\mathrm{RT}} \right)\frac{\left[ \mathrm{Na}^{+} \right]_{e}^{3}\left[ \mathrm{Ca}^{2+} \right]_{m}}{K_{\mathrm{Na}}^{3}K_{\mathrm{Ca}}}-\exp\left( \frac{-0.5{\Delta\Psi}_{m}F}{\mathrm{RT}} \right)\frac{\left[ \mathrm{Na}^{+} \right]_{m}^{3}\left[ \mathrm{Ca}^{2+} \right]_{i}}{K_{\mathrm{Na}}^{3}K_{\mathrm{Ca}}} \right)}{\left( 1+\frac{\left[ \mathrm{Na}^{+} \right]_{e}^{3}}{K_{\mathrm{Na}}^{3}}+\frac{\left[ \mathrm{Ca}^{2+} \right]_{m}}{K_{\mathrm{Ca}}}+\frac{\left[ \mathrm{Na}^{+} \right]_{e}^{3}\left[ \mathrm{Ca}^{2+} \right]_{m}}{K_{\mathrm{Na}}^{3}K_{\mathrm{Ca}}}+\frac{\left[ \mathrm{Na}^{+} \right]_{m}^{3}}{K_{\mathrm{Na}}^{3}}+\frac{\left[ \mathrm{Ca}^{2+} \right]_{i}}{K_{\mathrm{Ca}}}+\frac{\left[ \mathrm{Na}^{+} \right]_{m}^{3}\left[ \mathrm{Ca}^{2+} \right]_{i}}{K_{\mathrm{Na}}^{3}K_{\mathrm{Ca}}} \right)}$ ,

where $V_{\mathrm{NC}}$ is the Na^+^/Ca^2+^ exchanger maximal velocity, $K_{\mathrm{Ca}}$ is the Na^+^/Ca^2+^ exchanger Ca^2+^ affinity and $K_{\mathrm{Na}}$ is the Na^+^/Ca^2+^ exchanger Na^+^ affinity.

1. $\frac{d\left[ \mathrm{Ca}^{2+} \right]_{m}}{\mathrm{dt}}=\beta_{\mathrm{Ca}}\left( J_{\mathrm{uni}}-J_{\mathrm{NC}} \right)$
2. $\frac{d{\Delta\Psi}_{m}}{\mathrm{dt}}=\frac{V_{\mathrm{He}}+V_{He(F)}-V_{\mathrm{Hu}}-V_{\mathrm{ANT}}-V_{\mathrm{HLeak}}-I_{\mathrm{NaCa}}-2J_{\mathrm{uni}}}{C_{\mathrm{mito}}}$

**Table S7: Mitochondrial Ca^2+^ parameters**

| 1/ms | 2.159 | Uniporter Ca^2+^ permeability | P_Ca_ |
| --- | --- | --- | --- |
| [] | 2 | Ca^2+^ valence | Z_Ca_ |
| [] | 0.2 | Mitochondrial Ca^2+^ activity coefficient | _m_α |
| [] | 0.341 | Extramitochondrial Ca^2+^ activity coefficient | α_e_ |
| mM/ms | 1.863·10^-2^ | Na^+^/Ca^2+^ exchanger maximal velocity | V_NC_ |
| mM | 5 | Extramitochondrial Na^+^ concentration | Na_e_ |
| mM | 3.96 | Mitochondrial Na^+^ concentration | Na_e_ |
| [] | 1 | Ca^2+^ fraction that binds to Ca^2+^ buffers in the mitochondria | β_Ca_ |
| mM | 1.27·10^-3^ | KGDHC Ca^2+^ binding constant | K_D_^Ca^ |
| mM | 0.0308 | KGDHC Mg^2+^ binding constant | K_D_^Mg^ |
| mM/mV | 1.812·10^-3^ | Inner membrane capacitance | C_mito_ |

**References**

1. Lindblad, D.S., C.R. Murphey, J.W. Clark, and W.R. Giles. 1996. A model of the action potential and underlying membrane currents in a rabbit atrial cell. *Am. J. Physiol. - Hear. Circ. Physiol.* 271.

2. Hilgemann, D.W., and D. Noble. 1987. Excitation-contraction coupling and extracellular calcium transients in rabbit atrium: reconstruction of basic cellular mechanisms. *Proc. R. Soc. Lond. B. Biol. Sci.* 230:163–205.

3. Aslanidi, O. V., M.R. Boyett, H. Dobrzynski, J. Li, and H. Zhang. 2009. Mechanisms of transition from normal to reentrant electrical activity in a model of rabbit atrial tissue: Interaction of tissue heterogeneity and anisotropy. *Biophys. J.* 96:798–817.

4. Yaniv, Y., R. Sivan, and A. Landesberg. 2006. Stability, controllability, and observability of the “four state” model for the sarcomeric control of contraction. *Ann. Biomed. Eng.* 34.

5. Cortassa, S., M.A. Aon, B. O’Rourke, R. Jacques, H.J. Tseng, E. Marban, and R.L. Winslow. 2006. A computational model integrating electrophysiology, contraction, and mitochondrial bioenergetics in the ventricular myocyte. *Biophys J*. 91:1564–1589.

6. Harris, D.A., and A.M. Das. 1991. Control of mitochondrial ATP synthesis in the heart. *Biochem. J.* 280 ( Pt 3):561–573.

7. Chance, B., and G.R. Williams. 1955. A method for the localization of sites for oxidative phosphorylation. *Nature*. 176:250–254.

8. Nguyen, M.H., S.J. Dudycha, and M.S. Jafri. 2007. Effect of Ca2+ on cardiac mitochondrial energy production is modulated by Na+ and H+ dynamics. *Am J Physiol Cell Physiol*. 292:C2004-20.
